# Control of the HIV-1 Load Varies by Viral Subtype in a Large Cohort of African Adults With Incident HIV-1 Infection

**DOI:** 10.1093/infdis/jiz127

**Published:** 2019-04-02

**Authors:** Matt A Price, Wasima Rida, William Kilembe, Etienne Karita, Mubiana Inambao, Eugene Ruzagira, Anatoli Kamali, Eduard J Sanders, Omu Anzala, Eric Hunter, Susan Allen, Vinodh A Edward, Kristin M Wall, Jianming Tang, Patricia E Fast, Pontiano Kaleebu, Shabir Lakhi, Gaudensia Mutua, Linda Gail Bekker, Ggayi Abu-Baker, Amanda Tichacek, Paramesh Chetty, Mary H Latka, Pholo Maenetje, Heeran Makkan, Freddie Kibengo, Fran Priddy, Jill Gilmour

**Affiliations:** 1 International AIDS Vaccine Initiative, New York, New York; 2 Department of Epidemiology and Biostatistics, University of California–San Francisco, Atlanta, Georgia; 3 Rwanda Zambia HIV Research Group, Lusaka and Ndola; 4 Rwanda Zambia HIV Research Group, Zambia and Kigali; 5 Rwanda Zambia HIV Research Group, Rwanda; 6 Department of Epidemiology, and, Atlanta, Georgia; 7 Department of Pathology and Laboratory Medicine, Emory University, Atlanta, Georgia; 8 Department of Medicine, University of Alabama–Birmingham, New Haven, Connecticut; 9 Department of Environmental Health Sciences, Yale School of Public Health, New Haven, Connecticut; 10 MRC/UVRI Uganda Research Unit on AIDS, Entebbe, Uganda; 11 Kenyan Medical Research Institute–Wellcome Trust, Kilifi; 12 KAVI Institute of Clinical Research, Nairobi, Kenya; 13 Nuffield Department of Clinical Medicine, Centre for Clinical Vaccinology and Tropical Medicine, University of Oxford, Headington; 14 School of Pathology, Faculty of Health Sciences, University of the Witwatersrand; 15 Advancing Care and Treatment for TB/HIV, South African Medical Research Council, Johannesburg; 16 Desmond Tutu HIV Foundation, Cape Town, South Africa, Johannesburg; 18 Desmond Tutu HIV Foundation, Cape Town, South Africa

**Keywords:** HIV, AIDS, Africa, epidemiology, HIV subtype

## Abstract

Few human immunodeficiency virus (HIV)–infected persons can maintain low viral levels without therapeutic intervention. We evaluate predictors of spontaneous control of the viral load (hereafter, “viral control”) in a prospective cohort of African adults shortly after HIV infection. Viral control was defined as ≥2 consecutively measured viral loads (VLs) of ≤10 000 copies/mL after the estimated date of infection, followed by at least 4 subsequent measurements for which the VL in at least 75% was ≤10 000 copies/mL in the absence of ART. Multivariable logistic regression characterized predictors of viral control. Of 590 eligible volunteers, 107 (18.1%) experienced viral control, of whom 25 (4.2%) maintained a VL of 51–2000 copies/mL, and 5 (0.8%) sustained a VL of ≤50 copies/mL. The median ART-free follow-up time was 3.3 years (range, 0.3–9.7 years). Factors independently associated with control were HIV-1 subtype A (reference, subtype C; adjusted odds ratio [aOR], 2.1 [95% confidence interval {CI}, 1.3–3.5]), female sex (reference, male sex; aOR, 1.8 [95% CI, 1.1–2.8]), and having HLA class I variant allele B*57 (reference, not having this allele; aOR, 1.9 [95% CI, 1.0–3.6]) in a multivariable model that also controlled for age at the time of infection and baseline CD4^+^ T-cell count. We observed strong associations between infecting HIV-1 subtype, HLA type, and sex on viral control in this cohort. HIV-1 subtype is important to consider when testing and designing new therapeutic and prevention technologies, including vaccines.

## Teaser

Human immunodeficiency virus type 1 (HIV-1) subtype is important. We show that HIV subtype A is associated with viral control, using subtype C as a reference. Data from this and other studies suggest that HIV-1 subtype should be considered when designing new HIV therapeutic agents, prevention modalities, and vaccines.

Effective control of human immunodeficiency virus (HIV) infection is associated with improved clinical outcomes, including delayed onset of AIDS [1, 2], and studies indicate that the risk of sexually transmitting HIV is reduced for individuals with low plasma HIV loads [3, 4]. Spontaneous control of viral replication is rare, typically occurring in fewer than 5%–10% of all persons with HIV infection [1, 5]; it is also not well understood, particularly in the African context. A better understanding of spontaneous viral control could aid in development of new HIV treatment and cure options, prevention technologies, and vaccine design [6, 7].

The definition of HIV load control varies. All definitions include persons who are HIV seropositive by antibody testing (such as enzyme-linked immunosorbent assay [ELISA], rapid test, or Western blot) but who have a low or nondetectable HIV load in the absence of ART. A recent systematic review found that viremic controllers are most often defined as those with viral loads of <2000 copies/mL, but there was no clear consensus on the definition of control, and thresholds varied by up to 15 000 copies/mL [8]. Though our understanding of viral control is improving, a broader understanding of the role of the infecting viral subtype remains lacking. Here, we evaluate predictors of viral control in a cohort of volunteers from large, well-defined HIV-incidence cohorts in Africa enrolled within 1 year of their estimated date of infection.

## METHODS

### Ethical Considerations

This study was approved by local ethics review (Supplementary Materials). All volunteers provided informed consent before the collection of any study-related data.

### Recruitment and Study Procedures

Volunteers were primarily recruited from HIV-1 epidemiology studies at 9 clinical research centers in Kenya, Rwanda, South Africa, Uganda, and Zambia, as described elsewhere [9]; additional details are in the Supplementary Materials.

At enrollment, each volunteer’s medical history was recorded and a physical examination was conducted. Volunteers were followed monthly for the first 3 months after the estimated date of HIV infection, quarterly through 2 years after the estimated date of infection, and every 6 months thereafter. At each follow-up visit, volunteers were asked whether they had started ART or taken antiretroviral drugs for prevention of mother-to-child transmission of HIV. A symptoms-directed physical examination was performed. All volunteers were assessed for ART eligibility per the national guidelines and referred for treatment as appropriate. Blood specimens were collected for syphilis-specific serologic analysis (performed annually), measurement of CD4^+^ and CD8^+^ T-cell counts (by FACSCount or FACSCalibur; Becton-Dickinson Biosciences, Rapid plasma reagin, Biotec Laboratories, Inc., UK), and, if required, confirmatory HIV testing (eg, for volunteers who tested positive for HIV p24 antigen but negative for HIV antibodies at enrollment). Peripheral blood mononuclear cells were collected and stored; plasma was stored for viral load testing and subtype determination. The latter was derived from an amplified 1.7-kb segment of the *pol* gene, using the REGA HIV-1 subtyping tool and the Stanford database (available at: http://hivdb.stanford.edu/). If the subtype could not be assigned by using the REGA tool, additional phylogenetic analysis was done [10]. Genomic DNA samples derived from peripheral blood mononuclear cells were used for genotyping 3 HLA class I loci (*HLA-A*, *HLA-B*, and *HLA-C*), with individual alleles resolved to 4-digit specificities by using polymerase chain reaction–based techniques and current HLA nomenclatures [11].

### Definition of Viral Control

Viral control included aviremic control (viral load threshold, ≤ 50 copies/mL), viremic control (viral load threshold, 51–2000 copies/mL), and weak control (viral load threshold, 2001–10 000 copies/mL), collectively referred to as sustained viral control. Sustained viral control was defined as ≥2 consecutive viral load measurements ≤10 000 copies/mL (for weak control), ≤2000 copies/mL (for viremic control), and ≤50 copies/mL (for aviremic control) within 3–36 months after the estimated date of infection, plus at least 4 subsequent measurements for which the viral load in at least 75% was ≤10 000, ≤2000, and ≤50 copies/mL, respectively. All visits after the initiation of ART were excluded from analysis. No viral load data were recorded during short-term use of antiretroviral drugs for prevention of mother-to-child transmission of HIV. Plasma from participants was tested for the presence of antiretroviral drugs at the time their viral load dropped to ≤2000 copies/mL (Supplementary Materials).

### Data Analysis

Multivariable logistic regression analysis with the ART-free follow-up duration as an offset variable was used to characterize predictors of viral control. Missing data on HIV-1 subtype were imputed using the subtype of the participant’s suspected transmitting partner, if known. For participants in South Africa or Zambia, subtype C was assumed if no partner data were available.

All analyses were performed using R, version 3.4.3 (available at: https://www.R-project.org), using the package “glmulti,” to determine the best regression model among models with up to 6 covariates from a set of 26 covariates that included year of the estimated date of infection, age group at estimated date of infection (<25 years vs ≥25 years), sex, risk group, HIV-1 subtype, baseline CD4^+^ T-cell count, and HLA alleles present in ≥50 volunteers. Baseline CD4^+^ T-cell count was defined as the month 3 measurement or the average of measurements that fell within the window of the month 3 visit. Missing baseline CD4^+^ T-cell counts were imputed using chained equations in which the month 6 and month 12 CD4^+^ T-cell counts, along with age group (<25 years vs ≥25 years), sex, subtype, and geographic region, were used to model the missing data. No interaction terms were included, except for sex by A*03 when prior data [12] suggested women with this allele were more likely to control whereas men were not. The best model is defined as the model with the smallest value for the Akaike information criterion, a measure that balances a model’s simplicity with its fit of the data [13].

Six sensitivity analyses were performed (Supplementary Materials). The first limited the analysis to participants with at least 1 year of ART-free follow-up, to assess the effect of participants who withdrew from the study early. The second analysis adjusted for ART guidelines that varied over time and by country. For this analysis, the time to recommended ART initiation was calculated for each participant, based on their CD4^+^ T-cell count over time, and used an approximation to Rwanda’s ART guidelines (ie, the most-conservative guidelines). Follow-up time was then calculated as the time from the estimated date of infection to the earliest occurrence of the following: actual ART initiation, recommended ART initiation, or last study visit. The third sensitivity analysis replaced HIV-1 subtype with a subtype-by-geographic-region covariate in which sites were categorized as belonging to either eastern or southern Africa. While subtype C is predominant (nearly 100%) in southern Africa, there was greater subtype diversity among eastern African participants. Fourth, we limited our analysis to women only, to allow a better comparison to the study by Venner et al [14]. Fifth, we excluded volunteers for whom we had to impute the baseline CD4^+^ T-cell count. Finally, we performed a multivariable analysis with a more conservative definition of controller, using a threshold of 2000 copies/mL.

## RESULTS

### Study Population and Viral Control

**Figure 1.**
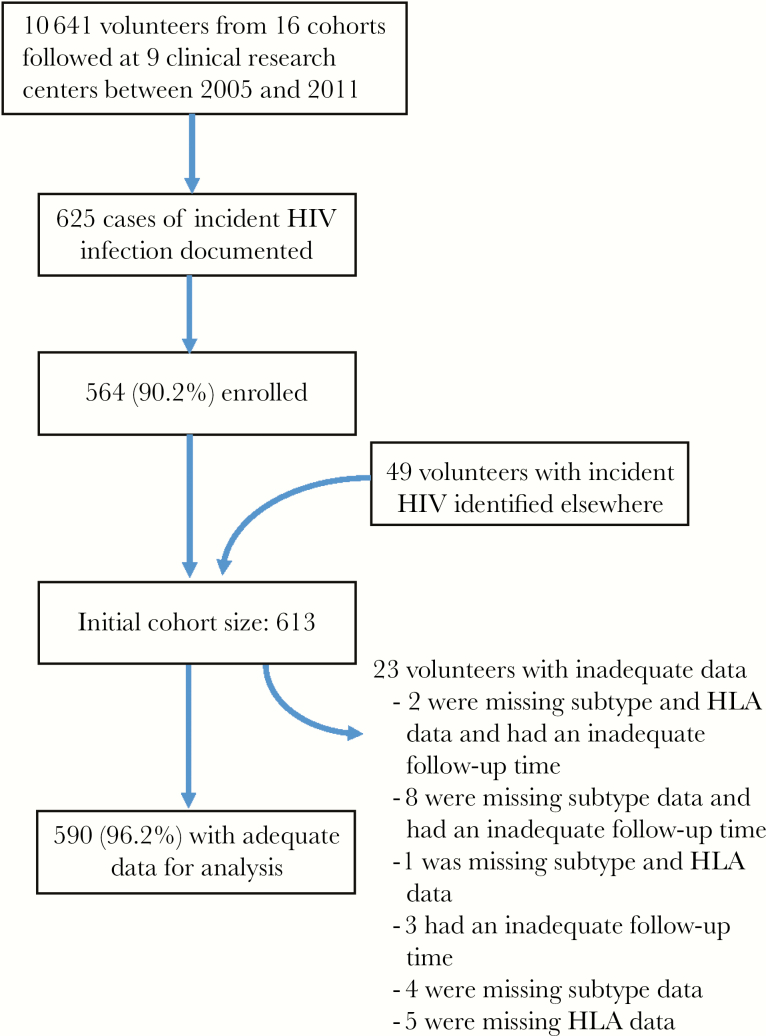
Flow of volunteers through the study. HIV, human immunodeficiency virus.

Between February 2006 and December 2011, 613 volunteers with incident HIV infection were identified and enrolled, including 113 from Kenya, 143 from Uganda, 94 from Rwanda, 234 from Zambia, and 29 from South Africa. Nearly all volunteers (564 [92%]) were identified during prospective follow-up in HIV incidence studies of key populations (Figure 1 and Table 1). Study follow-up data reported here were collected through April 2016.

**Table 1.** Description of Early Human Immunodeficiency Virus (HIV) Infection Cohort, by Location of Participating Clinical Research Center

Location	Study Start	Enrollment Complete	Source Population(s)	Enrolled, No.	Included in Analysis, No. (%)
Kigali, Rwanda	Feb 2006	May 2011	Discordant couples^a^	94	92 (97.9)
Masaka, Uganda	Jun 2006	Nov 2011	Rural communities, discordant couples^a^	97	95 (97.9)
Kilifi, Kenya	Jun 2006	Oct 2011	Walk-in VCT clients, FSW, MSM	88	84 (95.5)
Lusaka, Zambia	Jun 2006	Jul 2011	Discordant couples^a^	151	150 (99.3)
Kangemi, Kenya	Aug 2006	Jul 2010	FSW, clients of FSW, MSM	25	25 (100)
Entebbe, Uganda	Aug 2006	Oct 2010	Discordant couples,^a^ walk-in VCT clients	46	39 (84.8)
Ndola and Kitwe, Zambia	Oct 2006	Dec 2011	Discordant couples^a^	83	79 (95.2)
Cape Town, RSA	Dec 2006	Nov 2007	At-risk community members^b^	7	5 (71.4)
Rustenburg, RSA	Oct 2009	Dec 2011	At-risk community members,^b^ MSM	22	21 (95.5)
Summary	Feb 2006	Dec 2011	…	613	590 (96.2)

Abbreviations: FSW, female sex workers; MSM, men who report sex with men; RSA, Republic of South Africa; VCT, voluntary counseling and testing for HIV.

^a^Heterosexual cohabiting couples of discordant HIV status (one infected, one not).

^b^Self-reported heterosexual risk for HIV acquisition (see Methods).

Overall, 590 volunteers (96.2%) had sufficient data to evaluate viral control (Figure 1). Two hundred twenty-four volunteers (38.0%) achieved initial control (viral load, ≤10 000 copies/mL), but most later lost control of virus. In the cohort, 107 (18.1%) met our criteria for sustained viral control, with 25 (4.2%) and 5 (0.8%) exhibiting viremic and aviremic control, respectively. For the 107 controllers, the median time to control was 124 days (interquartile range, 84–302 days) after the estimated date of infection. The median follow-up time in the absence of ART was 3.3 years (range, 0.25–9.7 years). Over the course of follow-up, a similar proportion of controllers started ART, with 56.1% of viral controllers starting therapy in comparison to 59.4% of noncontrollers (*P* = .53). However, controllers had a longer median ART-free follow-up time than noncontrollers (5.1 years vs 3.2 years; *P* < .001, by the Wilcoxon rank sum test).

### Predictors of Viral Control

Table 2 presents baseline cohort characteristics by control status. The best multivariable model for viral control (Table 3) included baseline CD4^+^ T-cell count, sex, subtype, age at the estimated date of infection, and HLA alleles B*45:01, B*57, and B*58:02. Infection with HIV-1 subtype A as compared to subtype C was associated with control (adjusted odds ratio [aOR], 2.1 [95% confidence interval {CI}, 1.3–3.5]), as was having the HLA class I variant B*57 (aOR, 1.9 [95% CI, 1.0–3.6]). Women (aOR, 1.8 [95% CI, 1.1–2.8]) were also more likely to control than men (Table 3). Although not significant at the .05 level, the presence of a B*58:02 allele (aOR, 0.5 [95% CI, .3–1.0]; *P* = .08) appeared unfavorable with respect to control. Being older was also suggestive of a lower odds of control (aOR, 0.6 [95% CI, .4–1.0]; *P* = .07), and each 100-cell increase in a volunteer’s baseline CD4^+^ T-cell count was suggestive of an increased odds of control (aOR, 1.1 [95% CI, 1.0–1.2]; *P* = .06). The presence of a B*45:01 allele (aOR, 0.7 [95% CI, .3–1.3]; *P* = .22) was not associated with control status, although its inclusion in the model improved the fit of the data.

**Table 2.** Baseline Characteristics, by Human Immunodeficiency Virus (HIV) Control Status

Characteristic	Volunteers, No. (%)	Sustained Control, No. (%)^a^	Viremic Control, No. (%)^b^	Aviremic Control, No. (%)^c^
Overall	590 (100)	107 (18.4)	25 (4.2)	5 (0.9)
Study site				
Kigali, Rwanda	92 (100)	25 (27.2)	8 (8.7)	2 (2.2)
Masaka, Uganda	95 (100)	16 (16.8)	6 (6.3)	1 (1.1)
Kilifi, Kenya	84 (100)	18 (21.4)	3 (3.6)	0 (0)
Lusaka, Zambia	150 (100)	11 (7.3)	0 (0)	2 (1.3)
Kangemi, Kenya	25 (100)	7 (28.0)	5 (20.0)	0 (0)
Entebbe, Uganda	39 (100)	10 (25.6)	9 (23.1)	0 (0)
Ndola and Kitwe, Zambia	79 (100)	14 (17.7)	3 (3.8)	0 (0)
Cape Town, RSA	5 (100)	0 (0)	0 (0)	0 (0)
Rustenburg, RSA	21 (100)	6 (28.6)	2 (9.5)	0 (0)
Estimated time of infection				
2005–2006	170 (100)	25 (14.7)	10 (5.9)	1 (0.6)
2007–2008	218 (100)	41 (18.8)	7 (3.2)	3 (1.4)
2009–2011	202 (100)	41 (20.3)	8 (4.0)	1 (0.5)
Age at estimated time of infection				
<25 y	160 (100)	38 (23.8)	9 (5.6)	0 (0)
≥25 y	430 (100)	69 (16.1)	16 (3.7)	5 (1.2)
Sex and risk group				
Heterosexual male	261 (100)	33 (12.6)	8 (3.1)	2 (0.8)
MSM	90 (100)	17 (18.9)	2 (2.2)	0 (0)
Female	239 (100)	57 (23.9)	15 (6.3)	3 (1.3)
HLA-A*03, by sex				
Female				
No	213 (100)	49 (23.0)	12 (5.6)	1 (0.5)
Yes	26 (100)	8 (30.8)	3 (11.5)	2 (7.7)
Male				
No	325 (100)	46 (14.2)	9 (2.8)	2 (0.6)
Yes	26 (100)	4 (15.4)	1 (3.9)	0 (0)
HLA-B*45 allele				
No	499 (100)	96 (19.2)	22 (4.4)	5 (1.0)
Yes	91 (100)	11 (12.1)	3 (3.3)	0 (0)
HLA-B*58:02				
No	502 (100)	96 (19.1)	21 (4.2)	4 (0.8)
Yes	88 (100)	11 (12.5)	4 (4.6)	1 (1.1)
HLA-B*57				
No	535 (100)	90 (16.8)	19 (3.6)	4 (0.8)
Yes	55 (100)	17 (30.9)	6 (10.9)	1 (1.8)
HLA-B*81				
No	562 (100)	101 (18.0)	24 (4.3)	5 (0.9)
Yes	28 (100)	6 (21.4)	1 (3.6)	0 (0)
HIV-1 subtype				
C	273 (100)	38 (13.9)	6 (2.2)	2 (0.7)
A	207 (100)	52 (25.12)	15 (7.3)	3 (1.5)
D	81 (100)	15 (18.5)	4 (4.9)	0 (0)
Other^d^	29 (100)	2 (6.9)	0 (0)	0 (0)
Baseline CD4^+^ T-cell count^e^	533 (435–695)	646 (492–760)	712 (560–803)	483 (381–492)
ART-free follow-up duration, y^e^	3.3 (3.2–5.1)	5.1 (3.2–6.9)	6.0 (3.7–7.7)	6.9 (5.1, 6.9)

Data are no. of volunteers (% with characteristic), unless otherwise indicated.

Abbreviations: ART, antiretroviral therapy; MSM, men who report sex with men; RSA, Republic of South Africa.

^a^Volunteers who maintain a viral load of ≤10 000 copies/mL (ie, all viral controllers).

^b^Subset of sustained controllers who maintain a viral load of 51–2000 copies/mL.

^c^Subset of sustained controllers who maintain a viral load of ≤50 copies/mL.

^d^Twelve volunteers had subtype A1/D virus, 6 had subtype A1/C, 2 had subtype A1/A2/D, 2 had subtype CRF02_AG, 2 had subtype G, and 1 each had subtype A1/C/D, B, C/K, CRF11_CPX, or D/C.

^e^Data are median values (interquartile ranges) for 590 volunteers, 107 in the sustained control group, 25 in the viremic control group, and 5 in the aviremic control group.

**Table 3.** Association Between Baseline Covariates and Sustained Control of Human Immunodeficiency Virus (HIV) Load in an Early Infection Cohort

Covariate	Volunteers,^a^ No.	ART-Free Follow-Up, PY	Viral Controllers, No.	Unadjusted Analysis		Adjusted Analysis	
				OR (95% CI)	*P*	OR (95% CI)	*P*
Estimated time of infection							
2005–2006	170	760.5	25	Reference		…	
2007–2008	218	812.4	41	1.48 (.86–2.58)	.163	…	
2009–2011	202	570.2	41	1.82 (1.06–3.19)	.032	…	
Age at estimated time of infection							
<25 y	160	544.8	38	Reference		Reference	
≥25 y	430	1598.4	69	0.58 (.37–.91)	.016	0.64 (.39–1.06)	.068
Sex							
Male	351	1270.9	50	Reference		Reference	
Female	239	872.4	57	1.89 (1.24–2.90)	.003	1.78 (1.12–2.84)	.015
Risk group							
Discordant couple	427	1522.8	71	Reference			
MSM	90	311.7	17	1.19 (.64–2.12)	.556		
Other heterosexual	65	268	17	1.68 (.89–3.07)	.097		
Unknown	8	40.8	2	1.41 (.20–6.35)	.681		
HLA-A*03, by sex							
Female							
No	213	754.1	49	Reference			
Yes	26	118.3	8	1.33 (.51–3.18)	.539		
Male							
No	325	1166.7	46	0.55 (.35–.85)	.008		
Yes	26	104.2	4	0.57 (.16–1.59)	.326		
HLA-B*45:01							
No	499	1857.8	96	Reference		Reference	
Yes	91	285.5	11	0.62 (.3–1.16)	.160	0.65 (.31–1.25)	.222
HLA-B*58:02							
No	504	1819.2	96	Reference		Reference	
Yes	86	324	11	0.61 (.29–1.15)	.148	0.53 (.25–1.04)	.081
HLA-B*57							
No	535	1901.6	90	Reference		Reference	
Yes	55	241.6	17	2.02 (1.07–3.72)	.026	1.90 (.98–3.58)	.051
HLA-A*02:02							
No	535	1946.9	95	Reference			
Yes	55	196.3	12	1.32 (.64–2.55)	.424		
Baseline CD4^+^ T-cell count	590	2143.2	107	1.13^b^ (1.04–1.23)	.002	1.09^b^ (1.00–1.18)	.058
HIV-1 subtype							
C	273	929.7	38	Reference		Reference	
A	207	832.1	52	1.97 (1.24–3.17)	.004	2.09 (1.27–3.49)	.004
D	81	278.4	15	1.43 (.72–2.74)	.287	1.44 (.71–2.80)	.292
Other^c^	29	102.9	2	0.46 (.07–1.64)	.306	0.51 (.08–1.88)	.383

Abbreviations: ART, antiretroviral therapy; CI, confidence interval; MSM, men who report sex with men; OR, odds ratio; PY, person-years.

^a^Data are for 590 volunteers with viral load, subtype, and HLA data available.

^b^Data are the odds of viral control for every 100-cell increase in counts (ie, in the adjusted analysis, the odds of control increases 9% for every 100-cell increase in baseline CD4^+^ T-cell count).

^c^Twelve volunteers had subtype A1/D virus, 6 had subtype A1/C, 2 had subtype A1/A2/D, 2 had subtype CRF02_AG, 2 had subtype G, and 1 each had subtype A1/C/D, B, C/K, CRF11_CPX, or D/C.

### Aviremic and Viremic Viral Control

We observed too few aviremic and viremic viral controllers for a systematic multivariable analysis. However, after combining the aviremic with the viremic controllers, we found a significant association with HIV-1 subtype after adjustment for age group, sex, baseline CD4^+^ T-cell count, and presence of HLA allele B*57. With subtype C as the reference group, we observed an aOR of 3.31 (95% CI, 1.37–8.65) for subtype A and viral control (Supplementary Table 6).

### Sensitivity Analyses

While 5 of 6 analyses confirmed results of our primary analysis (Supplementary Materials), our analysis comparing controllers with subtype C infection in southern Africa to those in eastern Africa showed that East African volunteers with subtype C were more likely to control (aOR, 3.3 [95% CI, 1.1–9.1]) than southern African volunteers with subtype C infection. Because geography was significantly correlated with HIV subtype (98% of volunteers [249 of 255] in Zambia and South Africa were infected with subtype C), we were unable to initially control for it. The prevalence of control varied by site within southern Africa, ranging from no controllers in Cape Town to a prevalence of 7% in Lusaka, 18% in Copperbelt, and 29% in Rustenburg (*P* = .01, by the Fisher exact test; Table 2). This difference may be explained in part by the fact that Rustenburg volunteers were younger than volunteers from Zambia (median ages at the estimated date of infection, 25 and 33 years; *P* < .001, by the Wilcoxon rank sum test).

## DISCUSSION

In this adult, African cohort, we observed that 5% of volunteers demonstrated viral control to a level of ≤2000 viral copies/mL, including a small number (<1%) of aviremic controllers who maintained undetectable or nearly undetectable viral loads, while an additional 13% maintained weak control, with viral loads ranging from 2001 to 10 000 copies/mL. Subtype A–infected volunteers were more likely to control than subtype C–infected volunteers (aOR, 2.1 [95% CI, 1.3–3.5]), even when we controlled for multiple factors.

The definition of HIV load control varies across studies, making direct comparisons challenging. While we have presented our data according to the most common definitions observed in a systematic review of studies of viral control [8], owing to sample size limitations our multivariable analysis required the adoption of a broader definition of control. Most studies that report on viral control have done so in relatively homogeneous cohorts and rarely mention the infecting HIV-1 subtype. Okulicz et al, in a US military cohort, using the same viral thresholds as us, observed aviremic control in <1% of subjects and viremic control in 3% [1]. Madec et al found that 7% of seroconverters enrolled in CASCADE (comprising 22 cohorts across Canada, Europe, and Australia) also controlled virus to a level of <400/500 copies/mL at 2 consecutive visits, and this was more common among women than men [15]. Lambotte et al, in France, also used a different definition of control (<400 copies/mL in >90% of tested samples from individuals known to have been infected with HIV for >10 years) and found that control was exhibited in <1% of HIV-infected patients [5]. In an Australian cohort, the authors defined controllers as people with viral loads of <400 copies/mL for at least 2 years and observed a prevalence of 1.5% [2]. A recent prevalence study from Uganda defined controllers as persons who had a viral load of <2000 copies/mL, had a care duration of ≥5 years, were ART naive, and had a serial CD4^+^ T-cell count of ≥500 cells/µL; the authors reported a prevalence of 0.26%, although because limited data on their source population were shown, this is likely an underestimate [16].

In another recent study, Venner et al described a cohort of 286 African women in Uganda and Zimbabwe in which they observed a similar overall prevalence of viral control (ie, 7%, with control defined as a viral load of <2000 viral copies/mL and a CD4^+^ T-cell count >350 for >3 years). However, they observed the opposite correlation between subtype and control, noting that control was more common among women with HIV-1 subtype C infection as compared to those with subtype A infection [14]. While these results are contrary to ours, their cohort comprised only women, was smaller than ours, and did not control for HLA typing, which we found to be significantly associated with viral control and, as we have previously published, disease progression [17]. If we limited our analysis to the 239 women with HLA and subtype data (Supplementary Materials), we obtained results similar to our primary analysis when comparing subtype A to subtype C (aOR, 2.4 [95% CI, 1.1–5.2]). Furthermore, additional sensitivity analyses suggest that our observed relationship between infecting HIV-1 subtype and viral control persists under varying conditions. While Venner et al suggest that a hypothesized “milder” subtype C infection may be associated with a longer latency period, which in turn may allow for increased opportunities for transmission and thus greater spread, others have argued that the rapid spread of subtype C is due to higher replicative fitness and infectiousness [18, 19]. Our own work in this cohort has shown higher viral loads among persons infected with subtype C as compared to subtype A, supporting the hypothesis of subtype C’s increased infectiousness [20]. Results of epidemiologic studies have also been inconclusive. A report among African couples with a discordant HIV status (hereafter, HIV-discordant couples) reported no evidence of an increased risk of transmission associated with HIV-1 subtype C [21], while another study, from Uganda, found that HIV-1 subtype A has a higher rate of transmission than subtype D among HIV-discordant couples, but they were not able to assess subtype C [22]. Given that early treatment is important for improving clinical outcomes [23] and is increasingly common, observational studies such as these are no longer possible, and this question may never be answered definitively. However, as infecting subtype has been shown to be associated with clinical outcomes in multiple studies [14, 17, 24, 25] and now with viral control, it would seem prudent to consider how infecting subtype may affect new prevention technologies, including vaccines.

We evaluated a broad range of HLA alleles and found that HLA-B*57 was predictive of viremic control, although this association was of borderline significance (*P* = .051). This is in agreement with the literature on disease progression and viral control [26–29], although the relationships between B*57 and disease control often appear to be more robust, suggesting that this allele may play a stronger role in clinical outcomes than viral suppression. Previous studies have reported B*45 to be associated with poor clinical outcomes of HIV infection [30] or higher HIV loads [31], but here we found that volunteers with B*45:01 were not associated with control; however, the allele remained in our final model, suggesting that it may play some role in characterizing viral control. Some studies have suggested the presence of the B*58:02 allele to be unfavorable [32]; similarly, our data suggested that persons with the allele may be less likely to control HIV (aOR, 0.5 [95% CI, .2–1.0]). Previously, we observed in this cohort a sex-allele interaction with HLA-A*03, where women with A*03 were shown to have positive outcomes and men were not [12]. Although we observed a higher prevalence of viral control in women with A*03, with multivariable analysis we observed that this interaction was not predictive of viral control.

We found that women were more likely to be controllers than men. The CASCADE cohort found a similar result [15]. Route of exposure has been shown to affect viral fitness in the new host, with male-to-female transmission associated with lower viral fitness [33]. We have also previously shown that the sex of the seroconverter was associated with early set-point viral loads in HIV-discordant couples [34]. Less fit viruses may be easier to control, supporting our findings.

A limitation of this study is the relatively short follow-up duration for volunteers. Follow-up in many of the reports of viral control is measured in decades [1, 2, 5], and follow-up our study ranged from <1 year to nearly 10 years. We observed that some volunteers controlled virus only to lose control later (eg, see sensitivity analysis 2); with an increased follow-up duration, we may have observed more cases like this, lowering our estimated prevalence of control. Access to ART varied across country and region, introducing a bias in the length of ART-free follow-up by enrollment center. To control for this, we undertook a sensitivity analysis, using Rwandan government ART guidelines (Rwanda was typically the earliest adopter of new World Health Organization guidelines on ART initiation); standardizing our cohort to these guidelines, we still observed that control was more common among volunteers infected with subtype A. Our main finding of viral control being associated with HIV-1 subtype was driven in part by the low prevalence of viral control among volunteers from Lusaka, our largest recruitment center (Table 1). Our sensitivity analysis in which subtype was replaced by geographic region, suggested that eastern Africans infected with subtype C are more likely to control their virus than southern Africans and that the frequency of viral control between those with subtype A and those with subtype C in eastern Africa is similar (Supplementary Table 3). However, the number of eastern Africans infected with subtype C is small (n = 24), and our observed regional difference in viral control may be due to other unmeasured covariates.

Nearly one third of volunteers were enrolled too late to have a blood specimen collected at baseline (defined as 3 months after estimated date of HIV acquisition) for determination of the CD4^+^ T-cell count, and for these volunteers we imputed this data point. In another sensitivity analysis, we limited our analysis to the 392 volunteers with baseline CD4^+^ T-cell count data and found that the relationship between subtype A and subtype C persisted, although with the smaller sample size, the statistical significance was attenuated. Perhaps more importantly, we did not have CD4^+^ T-cell count estimates for volunteers before they acquired HIV infection. In a study conducted by these teams and in many of these clinical research centers, we observed a significant difference in CD4^+^ T-cell count, with lower counts observed among healthy, HIV-uninfected persons living in Zambia as compared to East Africa [35], similar to what Venner et al also noted: Zimbabwean women tended to have lower CD4^+^ T-cell counts than their Ugandan counterparts [14]. Although the CD4^+^ T-cell count approximately 3 months after infection was correlated with viral control in this cohort, it would have been informative to describe any immunological discrepancies prior to HIV acquisition.

Also, 23 volunteers (4%) from the cohort were missing infecting HIV-1 subtype data, missing HLA data, or did not have an adequate amount ART-free follow-up time to contribute to the study (Figure 1). While this represents only a small portion of our overall cohort, early ART initiation or loss to follow-up may be associated with disease progression, and our estimate of viral control may thus be a modest overestimate were these volunteers to be included. Additionally, our subtypes are derived from the *pol* sequence, and we may have missed some viral recombination. The clinical significance of this is unclear, and our derivation of subtype still proved to be significantly associated with viral control.

A main strength of this cohort is the large, well-defined source populations across regions where multiple HIV subtypes are prevalent. These vaccine-preparedness cohorts focused on persons at elevated risk of HIV acquisition (eg, men who report sex with men and female sex workers), but in many cases, an individual was considered to have an elevated risk because they were married to a person with HIV infection. Because we enrolled >90% of consecutively identified volunteers with incident HIV infection from these vaccine-preparedness studies of HIV transmission, our estimates of the prevalence of viral control are less likely to be further affected by selection bias beyond being a member of these key populations. Because HIV testing for these volunteers was done quarterly or monthly and included p24 antigen testing and polymerase chain reaction analysis, the between-test period during which HIV infection occurred was narrow, allowing us to estimate the date of HIV infection with relative precision. Previously, we noted that symptoms of acute retroviral syndrome were more common among volunteers with subtype A infection than among those with subtype C or D infection—highlighting potential subtype-mediated differences prior to the onset of viral control [36].

Infecting HIV-1 subtype is important. Controlling for volunteer HLA type, sex, and other characteristics, we observed that persons infected with HIV subtype A were significantly more likely to control virus than were persons with subtype C. Previous research in this cohort and others has shown that subtype is also associated with disease progression. While this question may never be answered conclusively, our data suggest that infecting HIV-1 subtype should be considered when designing new HIV therapeutic agents, prevention modalities, or vaccines.

## Supplementary Data

Supplementary materials are available at *The Journal of Infectious Diseases* online. Consisting of data provided by the authors to benefit the reader, the posted materials are not copyedited and are the sole responsibility of the authors, so questions or comments should be addressed to the corresponding author.

jiz127_suppl_Supplementary_Data

## Notes

***Author contributions.*** M. A. P. was responsible for conceptualization, methodology, formal analysis, data curation, original draft preparation, review, and editing. W. R. was responsible for methodology, formal analysis, data curation, original draft preparation, review, and editing. W. K., E. K., M. I., E. R., A. K., E. J. S., O. A, V. E., J. T., S. L., G. M., L. G. B., G. A.-B., P. C., M. H. L., P. M., H. M., and F. K. were responsible for investigation, data curation, review, and editing. E. H., S. A., and F. P. were responsible for conceptualization, investigation, data curation, review, and editing. K. M. W. was responsible for formal analysis, data curation, original draft preparation, review, and editing. P. F., P. K., and J. G. were responsible for conceptualization, methodology, investigation, data curation, review, and editing. A. T. was responsible for investigation, data curation and analysis, review, and editing.

***Acknowledgments***. We thank all of the volunteers who enrolled into the Early Infection Cohort study, without whom this work would have been impossible; the staff of each clinical research center and the International AIDS Vaccine Initiative (IAVI) staff in Johannesburg and in London at IAVI’s Human Immunology laboratory, based at Imperial College; the teams at Clinical Laboratory Services and University of the Witwatersrand in Johannesburg, South Africa, responsible for viral load testing and HIV sequence generation and analysis; the clinical data base management team at the Perinatal HIV Research Center in Johannesburg, for their hard work; Dr Eduard Acosta and his laboratory team at the University of Alabama–Birmingham and Dr Raymond Schinazi and his laboratory team at Emory University, for accommodating our requests to screen for the presence of antiretroviral drugs; and the Fogarty AIDS International Training in Research Program and the Emory Center for AIDS Research for support of selected authors.

***Financial support.*** This study was sponsored by the International AIDS Vaccine Initiative (IAVI), which in turn is funded by many donors, including the Bill and Melinda Gates Foundation, the Ministry of Foreign Affairs of Denmark, Irish Aid, the Ministry of Finance of Japan in partnership with The World Bank, the Ministry of Foreign Affairs of the Netherlands, the Norwegian Agency for Development Cooperation, the United Kingdom Department for International Development, and the US Agency for International Development (the full list of IAVI donors is available at: http://www.iavi.org).

***Potential conflicts of interest.*** In the role of sponsor, International AIDS Vaccine Initiative (IAVI) staff in collaboration with research partners wrote the study protocol, monitored study progress, conducted study analysis, and the primary author of this article (M. A. P.) is an IAVI employee. All other authors report no potential conflicts. All authors have submitted the ICMJE Form for Disclosure of Potential Conflicts of Interest. Conflicts that the editors consider relevant to the content of the manuscript have been disclosed.
