# Corrigendum

**DOI:** 10.1093/infdis/jiz462

**Published:** 2019-11-24

In “Control of the HIV-1 Load Varies by Viral Subtype in a Large Cohort of African Adults With Incident HIV-1 Infection” by Matt A. Price et al [J Infect Dis 2019; https://doi.org/10.1093/infdis/jiz127], in the originally published article, two author affiliations were incorrect. The affiliations should be as follows:

Vinodh A. Edward,^9,15,16,17^, Paramesh Chetty,^1,14^

^1^International AIDS Vaccine Initiative, New York, New York;

^9^Department of Environmental Health Sciences, Yale School of Public Health, New Haven, Connecticut;

^14^School of Pathology, Faculty of Health Sciences, University of the Witwatersrand, and

^15^Advancing Care and Treatment for TB/HIV, South African Medical Research Council, Johannesburg, and

^16^Desmond Tutu HIV Foundation, Cape Town, South Africa

^17^Advancing Care and Treatment for TB/HIV, South African Medical Research Council, Johannesburg.

