# Supplementary material for: Control of the HIV-1 Load Varies by Viral Subtype in a Large Cohort of African Adults With Incident HIV-1 Infection
Source: J Infect Dis. 2019 Apr 2;220(3):432–41. doi: 10.1093/infdis/jiz127 (PMC6603968; doi:10.1093/infdis/jiz127)
Supplement: jiz127_suppl_Supplementary_Data [file jiz127_suppl_supplementary_data.docx]

**Supplementary materials for Price et al. “HIV-1 viral control varies by viral subtype in a large cohort of African adults with incident HIV-1 infection”**

This document includes the following supplementary materials

1. Additional results from the five sensitivity analyses
   1. Analysis removing those volunteers with <1 year of ARV-drug-free follow up time
   2. Restricting follow up time to the time at which volunteers would be eligible for ARV drug initiation, per Rwandan Guidelines (the most conservative guidelines of the five countries that contributed volunteers)
   3. Include geography in the analysis, as geography was nearly perfectly collinear with HIV-1 subtype; all but one volunteer from southern africa (Zambia and South Africa) were infected with HIV-1 subtype C.
   4. Limiting analysis to women, to better allow comparisons with Venner et al., one of the only papers to look at similar outcomes to ours in an African cohort
   5. Restricting analysis to those volunteers with baseline CD4 T cell count. Baseline CD4 T cell count was defined as the Month 3 measurement or the average of measurements that fell within the window of the Month 3 visit (if there was more than one measurement taken)
   6. Multivariable analysis with more conservative definition of viral controllers (defined at threshold of ≤2,000c/mL instead of ≤10,000 c/mL)
2. Methods and additional details on the detection of HIV antiretroviral (ARV) therapy drugs used to detect volunteers with low viral loads who had not reported ARV
3. Details on ethical review
4. Details on source population cohorts, volunteer identification and calculation of estimated date of HIV infection
5. Genbank Accession Numbers for study volunteers with pol sequences for subtype determination.
6. **Additional results from the five sensitivity analyses**

Sensitivity Analysis 1: removing those volunteers with <1 year of ARV-drug-free follow up time. Eighty two (13.9%) volunteers had less than one year of ART free follow-up time. These volunteers did not differ by age, sex, or HIV-1 subtype. When we limited the analysis to the 508 volunteers with at least one year of ART free follow-up time, the best regression model was the same as that of our primary analysis with similar adjusted odds ratios for sex, subtype, and presence of a B*57 allele (Supplementary Table 1). The number of controllers was reduced from 107 to 104. This reduction reflects the small number of volunteers we categorized as viral controllers who had less than a year of follow-up. Subtype A infection compared to subtype C remained significantly associated with viral control (aOR 2.3 [1.4-3.9]).

| Supplementary Table 1. Association between baseline covariates and control of HIV viral load (≤ 10,000 copies/mL) in an early infection cohort with at least 12 months of ART free follow-up (n = 508) | | | | | | | | | | | |
| --- | --- | --- | --- | --- | --- | --- | --- | --- | --- | --- | --- |
|  |  |  |  | Unadjusted Analysis | | | | Adjusted Analysis | | | |
|  | Viral Control | | |  | 95% CI | |  |  | 95% CI | |  |
|  | n | PY | con | uOR | LL | UL | p-value | aOR OR | LL | UL | p-value |
| Year of EDI |  |  |  |  |  |  |  |  |  |  |  |
| 2005 - 2006 | 151 | 748.7 | 25 | Ref |  |  |  |  |  |  |  |
| 2007 - 2008 | 187 | 793.7 | 40 | 1.48 | 0.85 | 2.6 | 0.167 |  |  |  |  |
| 2009 - 2011 | 170 | 551.3 | 39 | 1.82 | 1.04 | 3.21 | 0.037 |  |  |  |  |
|  |  |  |  |  |  |  |  |  |  |  |  |
| Age at EDI |  |  |  |  |  |  |  |  |  |  |  |
| < 25 years | 136 | 531.4 | 37 | Ref |  |  |  | Ref |  |  |  |
| ≥ 25 years | 372 | 1562.3 | 67 | 0.56 | 0.35 | 0.89 | 0.014 | 0.62 | 0.38 | 1.02 | 0.055 |
|  |  |  |  |  |  |  |  |  |  |  |  |
| Sex |  |  |  |  |  |  |  |  |  |  |  |
| Male | 297 | 1237.6 | 48 | Ref |  |  |  | Ref |  |  |  |
| Female | 211 | 856 | 56 | 1.92 | 1.24 | 2.97 | 0.003 | 1.88 | 1.16 | 3.05 | 0.010 |
|  |  |  |  |  |  |  |  |  |  |  |  |
| Risk Group |  |  |  |  |  |  |  |  |  |  |  |
| Discordant Couple | 365 | 1484.5 | 68 | Ref |  |  |  |  |  |  |  |
| MSM | 76 | 303.9 | 17 | 1.27 | 0.68 | 2.28 | 0.436 |  |  |  |  |
| Other Heterosexual | 60 | 265.2 | 17 | 1.70 | 0.89 | 3.13 | 0.096 |  |  |  |  |
| Unknown | 7 | 40 | 2 | 1.48 | 0.21 | 7.06 | 0.644 |  |  |  |  |
|  |  |  |  |  |  |  |  |  |  |  |  |
| HLA A*02:02 |  |  |  |  |  |  |  |  |  |  |  |
| No | 459 | 1900.9 | 93 | Ref |  |  |  |  |  |  |  |
| Yes | 49 | 192.8 | 11 | 1.19 | 0.56 | 2.36 | 0.633 |  |  |  |  |
|  |  |  |  |  |  |  |  |  |  |  |  |
| HLA A*03 by sex |  |  |  |  |  |  |  |  |  |  |  |
| Female without | 188 | 740.6 | 49 | Ref |  |  |  |  |  |  |  |
| Female with | 23 | 115.5 | 7 | 1.12 | 0.41 | 2.80 | 0.821 |  |  |  |  |
| Male without | 274 | 1135.7 | 44 | 0.52 | 0.33 | 0.83 | 0.006 |  |  |  |  |
| Male with | 23 | 101.9 | 4 | 0.57 | 0.16 | 1.61 | 0.325 |  |  |  |  |
|  |  |  |  |  |  |  |  |  |  |  |  |
| HLA B*45:01 allele |  |  |  |  |  |  |  |  |  |  |  |
| No | 431 | 1816.9 | 93 | Ref |  |  |  | Ref |  |  |  |
| Yes | 77 | 276.8 | 11 | 0.65 | 0.31 | 1.23 | 0.211 | 0.68 | 0.32 | 1.33 | 0.283 |
|  |  |  |  |  |  |  |  |  |  |  |  |
| HLA B*57 |  |  |  |  |  |  |  |  |  |  |  |
| No | 458 | 1855.5 | 87 | Ref |  |  |  | Ref |  |  |  |
| Yes | 50 | 238.1 | 17 | 2.07 | 1.08 | 3.86 | 0.024 | 1.97 | 1.00 | 3.77 | 0.044 |
|  |  |  |  |  |  |  |  |  |  |  |  |
| HLA B*58:02 |  |  |  |  |  |  |  |  |  |  |  |
| No | 433 | 1776.2 | 93 | Ref |  |  |  | Ref |  |  |  |
| Yes | 75 | 317.5 | 11 | 0.62 | 0.30 | 1.18 | 0.168 | 0.54 | 0.25 | 1.07 | 0.095 |
|  |  |  |  |  |  |  |  |  |  |  |  |
| HIV-1 subtype |  |  |  |  |  |  |  |  |  |  |  |
| C | 236 | 906.2 | 36 | Ref |  |  |  | Ref |  |  |  |
| A | 182 | 816.6 | 52 | 2.11 | 1.31 | 3.44 | 0.002 | 2.30 | 1.38 | 3.90 | 0.002 |
| D | 68 | 271.1 | 14 | 1.43 | 0.70 | 2.81 | 0.306 | 1.48 | 0.71 | 2.95 | 0.282 |
| other | 22 | 99.8 | 2 | 0.51 | 0.08 | 1.86 | 0.379 | 0.57 | 0.09 | 2.14 | 0.467 |
|  |  |  |  |  |  |  |  |  |  |  |  |
| Month 3 CD4* count | 508 | 2093.7 | 104 | 1.11 | 1.02 | 1.21 | 0.016 | 1.06 | 0.96 | 1.16 | 0.224 |
|  |  |  |  |  |  |  |  |  |  |  |  |
| Total N = 508 volunteers with viral load, subtype, and HLA data and at least 12 months ART free follow-up. Includes 104 controllers (con). PY: person years of ART-free follow-up  MSM: Men who have sex with men  * Odds ratio represents the increase in odds of viral control for every 100-cell increase in the month 3 CD4 T cell count | | | | | | | | | | | |

Sensitivity Analysis 2: Restricting follow up time to the time at which volunteers would be eligible for ARV drug initiation, per Rwandan Guidelines (the most conservative guidelines of the five countries that contributed volunteers). Specifically, a participant was recommended for ART if: (1) his CD4 count was ≤ 200 after January 1, 2003; (2) his CD4 count was ≤ 350 after January 1, 2009; (3) his CD4 ≤ 500 after January 1, 2013; or (4) after January 1, 2016 when test and treat was recommended. By restricting follow-up time to the earliest of actual ART initiation, recommended ART initiation per Rwandan National Guidelines, or last study visit with viral load data, our sample size was nearly unchanged (n=589). One non-controlling volunteer from Zambia was excluded due to viral load data not being available prior to an early and very low CD4 count (< 200) that qualified him for ART under the Rwandan guidelines. This volunteer had persistently high viral loads (>100,000 copies/mL) and started ART about 14 months following HIV infection.

In this analysis, ART free follow-up time that incorporated the Rwandan guidelines was shorter with a median (IQR) of 1.8 years (0.9 – 3.3 years). Of the 107 volunteers classified as controllers in the primary analysis, 13 were re-classified as non-controllers when censored for ART initiation under Rwandan guidelines. Of the 483 volunteers classified as non-controllers, 17 were re-classified as controllers. These volunteers tended to have longer follow-up whereby they initially controlled their virus, but then lost control in the absence of ART. By censoring them earlier, they are more likely to be considered controllers. Thus, the number of volunteers exhibiting sustained control increased from 107 to 111. The best regression model was the same as that of the primary analysis (Supplementary Table 2) except sex was replaced by HLA A*03 by sex. Subtype A infected volunteers were more likely to control than subtype C infected volunteers (aOR: 2.7 [1.6-4.5]).

| Supplemental Table 2. Association between baseline covariates and control of HIV viral load (≤ 10,000 copies/mL) in an early infection cohort with adjusted ART free follow-up time employing Rwandan National ART Guidelines (n = 589) | | | | | | | | | | | | |  |
| --- | --- | --- | --- | --- | --- | --- | --- | --- | --- | --- | --- | --- | --- |
|  |  |  |  | Unadjusted Analysis | | | | Adjusted Analysis | | | | |  |
|  | Viral Control | | |  | 95% CI | |  | |  | 95% CI | |  | |
|  | n | PY | con | uOR | LL | UL | p-value | | aOR | LL | UL | p-value | |
| Year of EDI |  |  |  |  |  |  |  | |  |  |  |  | |
| 2005 - 2006 | 170 | 543.8 | 30 | Ref |  |  |  | |  |  |  |  | |
| 2007 - 2008 | 217 | 511.4 | 41 | 1.28 | 0.76 | 2.18 | 0.354 | |  |  |  |  | |
| 2009 - 2011 | 202 | 337.3 | 40 | 1.61 | 0.95 | 2.75 | 0.078 | |  |  |  |  | |
|  |  |  |  |  |  |  |  | |  |  |  |  | |
| Age at EDI |  |  |  |  |  |  |  | |  |  |  |  | |
| < 25 years | 160 | 358.7 | 38 | Ref |  |  |  | | Ref |  |  |  | |
| ≥ 25 years | 429 | 1033.9 | 73 | 0.62 | 0.4 | 0.98 | 0.038 | | 0.69 | 0.43 | 1.12 | 0.130 | |
|  |  |  |  |  |  |  |  | |  |  |  |  | |
| Sex |  |  |  |  |  |  |  | |  |  |  |  | |
| Male | 350 | 779.5 | 54 | Ref |  |  |  | |  |  |  |  | |
| Female | 239 | 613.1 | 57 | 1.63 | 1.07 | 2.48 | 0.023 | |  |  |  |  | |
|  |  |  |  |  |  |  |  | |  |  |  |  | |
| Risk Group |  |  |  |  |  |  |  | |  |  |  |  | |
| Discordant Couple | 426 | 1003.8 | 73 | Ref |  |  |  | |  |  |  |  | |
| MSM | 90 | 190 | 20 | 1.5 | 0.84 | 2.61 | 0.156 | |  |  |  |  | |
| Other Heterosexual | 65 | 171.8 | 15 | 1.45 | 0.74 | 2.69 | 0.258 | |  |  |  |  | |
| Unknown | 8 | 26.9 | 3 | 2.45 | 0.49 | 10.38 | 0.232 | |  |  |  |  | |
|  |  |  |  |  |  |  |  | |  |  |  |  | |
| HLA A*02:02 |  |  |  |  |  |  |  | |  |  |  |  | |
| No | 534 | 1261.1 | 97 | Ref |  |  |  | |  |  |  |  | |
| Yes | 55 | 131.5 | 14 | 1.52 | 0.77 | 2.87 | 0.206 | |  |  |  |  | |
|  |  |  |  |  |  |  |  | |  |  |  |  | |
| HLA A*03 by sex |  |  |  |  |  |  |  | |  |  |  |  | |
| Female without | 213 | 525.7 | 46 | Ref |  |  |  | | Ref |  |  |  | |
| Female with | 26 | 87.4 | 11 | 2.38 | 0.99 | 5.60 | 0.047 | | 2.43 | 0.95 | 6.04 | 0.058 | |
| Male without | 324 | 719.2 | 50 | 0.69 | 0.44 | 1.08 | 0.107 | | 0.72 | 0.44 | 1.18 | 0.189 | |
| Male with | 26 | 60.3 | 4 | 0.68 | 0.19 | 1.90 | 0.495 | | 0.68 | 0.17 | 2.12 | 0.531 | |
|  |  |  |  |  |  |  |  | |  |  |  |  | |
| HLA B*4501 allele |  |  |  |  |  |  |  | |  |  |  |  | |
| No | 498 | 1224.9 | 101 | Ref |  |  |  | | Ref |  |  |  | |
| Yes | 91 | 167.7 | 10 | 0.55 | 0.26 | 1.05 | 0.089 | | 0.58 | 0.27 | 1.15 | 0.140 | |
|  |  |  |  |  |  |  |  | |  |  |  |  | |
| HLA B*57 |  |  |  |  |  |  |  | |  |  |  |  | |
| No | 535 | 1242.6 | 93 | Ref |  |  |  | | Ref |  |  |  | |
| Yes | 54 | 149.9 | 18 | 2.21 | 1.17 | 4.05 | 0.012 | | 2.08 | 1.07 | 3.93 | 0.027 | |
|  |  |  |  |  |  |  |  | |  |  |  |  | |
| HLA B*5802 |  |  |  |  |  |  |  | |  |  |  |  | |
| No | 503 | 1175.3 | 98 | Ref |  |  |  | | Ref |  |  |  | |
| Yes | 86 | 217.3 | 13 | 0.71 | 0.36 | 1.30 | 0.290 | | 0.58 | 0.28 | 1.12 | 0.118 | |
|  |  |  |  |  |  |  |  | |  |  |  |  | |
| HIV-1 subtype |  |  |  |  |  |  |  | |  |  |  |  | |
| C | 272 | 564.8 | 34 | Ref |  |  |  | | Ref |  |  |  | |
| A | 207 | 559.9 | 58 | 2.52 | 1.58 | 4.09 | <0.001 | | 2.65 | 1.60 | 4.45 | <0.001 | |
| D | 81 | 207 | 17 | 1.73 | 0.89 | 3.29 | 0.098 | | 1.63 | 0.81 | 3.20 | 0.163 | |
| other | 29 | 60.9 | 2 | 0.53 | 0.08 | 1.91 | 0.405 | | 0.59 | 0.09 | 2.19 | 0.492 | |
|  |  |  |  |  |  |  |  | |  |  |  |  | |
| Month 3 CD4* | 589 | 1392.6 | 111 | 1.13 | 1.05 | 1.23 | 0.002 | | 1.09 | 1.00 | 1.19 | 0.043 | |
|  |  |  |  |  |  |  |  | |  |  |  |  | |
| Total N = 589 volunteers with viral load, subtype, and HLA data. Includes 114 controllers (Con) PY : person years of ART-free follow-up  MSM: Men who have sex with men  * Odds ratio represents the increase in odds of viral control for every 100-cell increase in the month 3 CD4 T cell count | | | | | | | | | | | | |  |

Sensitivity Analysis 3: Include geography in the analysis, as geography was nearly perfectly collinear with HIV-1 subtype; all but two volunteers from southern africa (Zambia and South Africa) were infected with HIV-1 subtype C. Given this confounding of subtype with geographic region, we generated a seven-level categorical variable based on a volunteer’s HIV-1 subtype and whether he/she resided in eastern or southern Africa. Our primary intent was to compare the 249 subtype C infected individuals living in southern Africa with the 24 subtype C volunteers in eastern Africa, as we had observed a different prevalence of control by region (12.5% sustained control in southern Africa vs. 29.2% sustained controllers in east Africa). A comparison of subtypes A and D across regions is not possible due to only 2 subtype A and 0 subtype D infections among southern African volunteers. We do observe that subtype C infected volunteers in east Africa appear to be more likely to control virus than those in southern Africa. After adjusting for age group, baseline CD4, sex, and presence of B*45:01, B*57, and B*58:02 alleles, we obtain an adjusted odds ratio of 3.3 [1.1-9.1] when comparing subtype C infected eastern Africans to subtype C infected southern Africans (Supplemental Table 3). Our association between subtype A infected volunteers and subtype C infected volunteers from southern Africa also persists (aOR: 2.5 [1.5-4.3]). Although the number of subtype C infections among eastern Africans is small in our study, this result raises the question of whether there are other, unmeasured regional differences beyond subtype at play.

| Supplemental Table 3. Association between baseline covariates and control of HIV viral load (≤ 10,000 copies/mL) in an early infection cohort including a subtype by region covariate (n = 590) | | | | | | | | | | | |
| --- | --- | --- | --- | --- | --- | --- | --- | --- | --- | --- | --- |
|  |  |  |  | Unadjusted Analysis | | | | Adjusted Analysis | | | |
|  | Viral Control | | |  | 95% CI | |  |  | 95% CI | |  |
|  | n | PY | con | uOR | LL | UL | p-value | aOR | LL | UL | p-value |
| Year of EDI |  |  |  |  |  |  |  |  |  |  |  |
| 2005 - 2006 | 170 | 760.5 | 25 | Ref |  |  |  |  |  |  |  |
| 2007 - 2008 | 218 | 812.4 | 41 | 1.48 | 0.86 | 2.58 | 0.163 |  |  |  |  |
| 2009 - 2011 | 202 | 570.2 | 41 | 1.82 | 1.06 | 3.19 | 0.032 |  |  |  |  |
|  |  |  |  |  |  |  |  |  |  |  |  |
| Age at EDI |  |  |  |  |  |  |  |  |  |  |  |
| < 25 years | 160 | 544.8 | 38 | Ref |  |  |  | Ref |  |  |  |
| ≥ 25 years | 430 | 1598.4 | 69 | 0.58 | 0.37 | 0.91 | 0.016 | 0.68 | 0.42 | 1.11 | 0.115 |
|  |  |  |  |  |  |  |  |  |  |  |  |
| Sex |  |  |  |  |  |  |  |  |  |  |  |
| Male | 351 | 1270.9 | 50 | Ref |  |  |  | Ref |  |  |  |
| Female | 239 | 872.4 | 57 | 1.89 | 1.24 | 2.90 | 0.003 | 1.96 | 1.22 | 3.17 | 0.005 |
|  |  |  |  |  |  |  |  |  |  |  |  |
| Risk Group |  |  |  |  |  |  |  |  |  |  |  |
| Discordant Couple | 427 | 1522.8 | 71 | Ref |  |  |  |  |  |  |  |
| MSM | 90 | 311.7 | 17 | 1.19 | 0.64 | 2.12 | 0.556 |  |  |  |  |
| Other Heterosexual | 65 | 268 | 17 | 1.68 | 0.89 | 3.07 | 0.097 |  |  |  |  |
| Unknown | 8 | 40.8 | 2 | 1.41 | 0.20 | 6.35 | 0.681 |  |  |  |  |
|  |  |  |  |  |  |  |  |  |  |  |  |
| HLA A*02:02 |  |  |  |  |  |  |  |  |  |  |  |
| No | 535 | 1946.9 | 95 | Ref |  |  |  |  |  |  |  |
| Yes | 55 | 196.3 | 12 | 1.32 | 0.64 | 2.55 | 0.424 |  |  |  |  |
|  |  |  |  |  |  |  |  |  |  |  |  |
| HLA A*03 by sex |  |  |  |  |  |  |  |  |  |  |  |
| Female - No | 213 | 754.1 | 49 | Ref |  |  |  |  |  |  |  |
| Female - Yes | 26 | 118.3 | 8 | 1.33 | 0.51 | 3.18 | 0.539 |  |  |  |  |
| Male - No | 325 | 1166.7 | 46 | 0.55 | 0.35 | 0.85 | 0.008 |  |  |  |  |
| Male - Yes | 26 | 104.2 | 4 | 0.57 | 0.16 | 1.59 | 0.326 |  |  |  |  |
|  |  |  |  |  |  |  |  |  |  |  |  |
| HLA B*45:01 |  |  |  |  |  |  |  |  |  |  |  |
| No | 499 | 1857.8 | 96 | Ref |  |  |  | Ref |  |  |  |
| Yes | 91 | 285.5 | 11 | 0.62 | 0.3 | 1.16 | 0.160 | 0.64 | 0.3 | 1.25 | 0.215 |
|  |  |  |  |  |  |  |  |  |  |  |  |
| HLA B*57 |  |  |  |  |  |  |  |  |  |  |  |
| No | 535 | 1901.6 | 90 | Ref |  |  |  | Ref |  |  |  |
| Yes | 55 | 241.6 | 17 | 2.02 | 1.07 | 3.72 | 0.026 | 1.97 | 1.01 | 3.76 | 0.041 |
|  |  |  |  |  |  |  |  |  |  |  |  |
| HLA B*58:02 |  |  |  |  |  |  |  |  |  |  |  |
| No | 504 | 1819.2 | 96 | Ref |  |  |  | Ref |  |  |  |
| Yes | 86 | 324 | 11 | 0.61 | 0.29 | 1.15 | 0.148 | 0.54 | 0.25 | 1.05 | 0.083 |
|  |  |  |  |  |  |  |  |  |  |  |  |
| Month 3 CD4* | 590 | 2143.2 | 107 | 1.13 | 1.04 | 1.23 | 0.002 | 1.08 | 0.99 | 1.18 | 0.063 |
| HIV-1 subtype by region |  |  |  |  |  |  |  |  |  |  |  |
| C – southern Africa | 249 | 831.6 | 31 | Ref |  |  |  | Ref |  |  |  |
| C – eastern Africa | 24 | 98.2 | 7 | 2.67 | 0.96 | 6.79 | 0.047 | 3.30 | 1.12 | 9.06 | 0.024 |
| A – southern Africa | 2 | 2.8 | 0 | 0.00 |  |  | 0.990 | 0.00 |  |  | 0.989 |
| A – eastern Africa | 205 | 829.4 | 52 | 2.25 | 1.38 | 3.72 | 0.001 | 2.51 | 1.48 | 4.33 | 0.001 |
| D – eastern Africa | 81 | 278.4 | 15 | 1.62 | 0.80 | 3.15 | 0.166 | 1.67 | 0.81 | 3.32 | 0.154 |
| Other – southern Africa | 4 | 13.1 | 0 | 0.00 |  |  | 0.985 | 0.00 |  |  | 0.985 |
| Other – eastern Africa | 25 | 89.8 | 2 | 0.61 | 0.09 | 2.23 | 0.520 | 0.71 | 0.11 | 2.69 | 0.660 |
| Total N = 590 volunteers with viral load, subtype, and HLA data, including 107 controllers (con)  PY = person years of ART-free follow-up  MSM: Men who have sex with men  * Odds ratio represents the increase in odds of viral control for every 100-cell increase in the month 3 CD4 T cell count | | | | | | | | | | | |

Sensitivity Analysis 4: Limiting analysis to women, to better allow comparisons with Venner et al., one of the only papers to look at similar outcomes to ours in an African cohort. Two hundred thirty nine (40.5%) volunteers were female. When the analysis was limited to females, the best regression model was based on 57 sustained controllers and included baseline CD4, subtype, and presence of alleles B*45:01, B*57, B*58:02, and A*02:02 (Supplemental Table 4). The final model, with age group included, showed a strong association between subtype and viral control. Subtype A infected women were more likely to control than their subtype C infected sisters (aOR: 2.4 [1.1-5.3]). In addition to the presence of B*57 being beneficial, the presence of A*02:02 appears favorable (aOR: 6.0 [1.6-25.9]). However, this association should be interpreted cautiously as only 17 women had the allele. Also, in a *post hoc* analysis limited to male volunteers, no association with A*02:02 was found (data not shown).

| Supplemental Table 4. Association between baseline covariates and control of HIV viral load (≤ 10,000 copies/mL) in an early infection cohort, females only (n = 239) | | | | | | | | | | | |
| --- | --- | --- | --- | --- | --- | --- | --- | --- | --- | --- | --- |
|  |  |  |  | Unadjusted Analysis | | | | Adjusted Analysis | | | |
|  | Viral Control | | |  | 95% CI | |  |  | 95% CI | |  |
|  | n | WY | con | uOR | LL | UL | p-value | aOR | LL | UL | p-value |
| Year of EDI |  |  |  |  |  |  |  |  |  |  |  |
| 2005 - 2006 | 72 | 318.5 | 12 | Ref |  |  |  |  |  |  |  |
| 2007 - 2008 | 91 | 350.6 | 26 | 2.13 | 1.00 | 4.75 | 0.056 |  |  |  |  |
| 2009 - 2011 | 76 | 203.3 | 19 | 2.10 | 0.94 | 4.84 | 0.075 |  |  |  |  |
|  |  |  |  |  |  |  |  |  |  |  |  |
| Age at EDI |  |  |  |  |  |  |  |  |  |  |  |
| < 25 years | 87 | 280.3 | 25 | Ref |  |  |  | Ref |  |  |  |
| ≥ 25 years | 152 | 592.1 | 32 | 0.59 | 0.32 | 1.09 | 0.09 | 0.62 | 0.31 | 1.21 | 0.157 |
|  |  |  |  |  |  |  |  |  |  |  |  |
| Risk Group |  |  |  |  |  |  |  |  |  |  |  |
| DC | 191 | 686.3 | 43 | Ref |  |  |  |  |  |  |  |
| Unknown | 3 | 14.1 | 1 | 1.60 | 0.07 | 18.00 | 0.708 |  |  |  |  |
| Other Heterosexual | 45 | 171.9 | 13 | 1.37 | 0.64 | 2.81 | 0.404 |  |  |  |  |
|  |  |  |  |  |  |  |  |  |  |  |  |
| HLA A*02:02 |  |  |  |  |  |  |  |  |  |  |  |
| No | 222 | 809.8 | 49 | Ref |  |  |  | Ref |  |  |  |
| Yes | 17 | 62.5 | 8 | 3.24 | 1.14 | 9.03 | 0.024 | 5.98 | 1.58 | 25.89 | 0.010 |
|  |  |  |  |  |  |  |  |  |  |  |  |
| HLA A*03 |  |  |  |  |  |  |  |  |  |  |  |
| No | 213 | 754.1 | 49 | Ref |  |  |  |  |  |  |  |
| Yes | 26 | 118.3 | 8 | 1.33 | 0.51 | 3.18 | 0.539 |  |  |  |  |
|  |  |  |  |  |  |  |  |  |  |  |  |
|  |  |  |  |  |  |  |  |  |  |  |  |
| HLA B*45:01 |  |  |  |  |  |  |  |  |  |  |  |
| No | 210 | 797 | 54 | Ref |  |  |  | Ref |  |  |  |
| Yes | 29 | 75.3 | 3 | 0.39 | 0.09 | 1.18 | 0.139 | 0.41 | 0.09 | 1.31 | 0.177 |
|  |  |  |  |  |  |  |  |  |  |  |  |
| HLA B*57 |  |  |  |  |  |  |  |  |  |  |  |
| No | 215 | 785.1 | 46 | Ref |  |  |  | Ref |  |  |  |
| Yes | 24 | 87.2 | 11 | 3.13 | 1.29 | 7.54 | 0.011 | 3.09 | 1.17 | 8.09 | 0.021 |
|  |  |  |  |  |  |  |  |  |  |  |  |
| HLA B*58:02 |  |  |  |  |  |  |  |  |  |  |  |
| No | 205 | 739.4 | 52 | Ref |  |  |  | Ref |  |  |  |
| Yes | 34 | 133 | 5 | 0.48 | 0.16 | 1.22 | 0.153 | 0.19 | 0.04 | 0.65 | 0.017 |
|  |  |  |  |  |  |  |  |  |  |  |  |
| HIV-1 subtype |  |  |  |  |  |  |  |  |  |  |  |
| C | 127 | 428.4 | 25 | Ref |  |  |  | Ref |  |  |  |
| A | 66 | 285.7 | 23 | 1.98 | 1.00 | 3.89 | 0.048 | 2.43 | 1.14 | 5.25 | 0.022 |
| D | 38 | 134.8 | 8 | 1.09 | 0.42 | 2.61 | 0.849 | 1.18 | 0.43 | 3.01 | 0.740 |
| other | 8 | 23.5 | 1 | 0.62 | 0.03 | 3.82 | 0.669 | 0.84 | 0.04 | 5.40 | 0.876 |
|  |  |  |  |  |  |  |  |  |  |  |  |
| Month 3 CD4* | 239 | 872.4 | 57 | 1.13 | 1.02 | 1.27 | 0.022 | 1.12 | 1.00 | 1.25 | 0.054 |
|  |  |  |  |  |  |  |  |  |  |  |  |
| Total N = 239 female volunteers with viral load, subtype, and HLA data, including 57 controllers (con)  WY = woman years of ART-free follow-up  * Odds ratio represents the increase in odds of viral control for every 100-cell increase in the month 3 CD4 T cell count | | | | | | | | | | | |

Sensitivity Analysis 5: Restricting analysis to those volunteers with baseline CD4 T cell count. Baseline CD4 T cell count was defined as the Month 3 measurement or the average of measurements that fell within the window of the Month 3 visit (if there was more than one measurement taken). One hundred ninety-eight (33.6%) volunteers did not have a baseline CD4 count primarily due to delayed enrollment into the study. These volunteers did not differ by age, sex, or HIV-1 subtype. Restricting our analysis to the 392 volunteers with a baseline CD4 count, we observe 72 sustained controllers (Supplemental Table 5). After accounting for age group, baseline CD4, sex, and presence of B*45:01, B*57, and B*58:02 alleles, the adjusted OR comparing subtype A to subtype C was 1.9 [1.0-3.7] with p = 0.042.

| Supplemental Table 5. Association between baseline covariates and control of HIV viral load (≤10,000 copies/ mL) in an early infection cohort, including only those volunteers with month 3 CD4 T cell counts (n = 392) | | | | | | | | | | | |
| --- | --- | --- | --- | --- | --- | --- | --- | --- | --- | --- | --- |
|  |  |  |  | Unadjusted Analysis | | | | Adjusted Analysis | | | |
|  | Viral Control | | |  | 95% CI | |  |  | 95% CI | |  |
|  | n | PY | con | uOR | LL | UL | p-value | aOR | LL | UL | p-value |
| Year of EDI |  |  |  |  |  |  |  |  |  |  |  |
| 2005 - 2006 | 54 | 235.3 | 11 | Ref |  |  |  |  |  |  |  |
| 2007 - 2008 | 170 | 597.3 | 30 | 0.9 | 0.42 | 2.03 | 0.795 |  |  |  |  |
| 2009 - 2011 | 168 | 475.7 | 31 | 1.04 | 0.49 | 2.34 | 0.92 |  |  |  |  |
|  |  |  |  |  |  |  |  |  |  |  |  |
| Age at EDI |  |  |  |  |  |  |  |  |  |  |  |
| < 25 years | 98 | 298.8 | 19 | Ref |  |  |  | Ref |  |  |  |
| ≥ 25 years | 294 | 1009.4 | 53 | 0.85 | 0.48 | 1.56 | 0.582 | 1.00 | 0.54 | 1.90 | 0.998 |
|  |  |  |  |  |  |  |  |  |  |  |  |
| Sex |  |  |  |  |  |  |  |  |  |  |  |
| Male | 236 | 787.9 | 34 | Ref |  |  |  | Ref |  |  |  |
| Female | 156 | 520.4 | 38 | 1.93 | 1.15 | 3.25 | 0.013 | 1.77 | 0.99 | 3.17 | 0.055 |
|  |  |  |  |  |  |  |  |  |  |  |  |
| Risk Group |  |  |  |  |  |  |  |  |  |  |  |
| Discordant Couple | 293 | 967 | 51 | Ref |  |  |  |  |  |  |  |
| MSM | 61 | 195.9 | 12 | 1.19 | 0.57 | 2.35 | 0.633 |  |  |  |  |
| Other Heterosexual | 34 | 123.7 | 8 | 1.42 | 0.57 | 3.23 | 0.422 |  |  |  |  |
| Unknown | 4 | 21.7 | 1 | 1.21 | 0.06 | 9.75 | 0.870 |  |  |  |  |
|  |  |  |  |  |  |  |  |  |  |  |  |
| HLA A*02:02 |  |  |  |  |  |  |  |  |  |  |  |
| No | 357 | 1188.2 | 65 | Ref |  |  |  |  |  |  |  |
| Yes | 35 | 120.1 | 7 | 1.11 | 0.43 | 2.54 | 0.821 |  |  |  |  |
|  |  |  |  |  |  |  |  |  |  |  |  |
| HLA A*03 by sex |  |  |  |  |  |  |  |  |  |  |  |
| Female - No | 139 | 455.2 | 32 | Ref |  |  |  |  |  |  |  |
| Female - Yes | 17 | 65.2 | 6 | 1.71 | 0.55 | 4.92 | 0.334 |  |  |  |  |
| Male - No | 220 | 737.7 | 33 | 0.58 | 0.34 | 1.00 | 0.051 |  |  |  |  |
| Male - Yes | 16 | 50.1 | 1 | 0.22 | 0.01 | 1.17 | 0.154 |  |  |  |  |
|  |  |  |  |  |  |  |  |  |  |  |  |
| HLA B*45:01 |  |  |  |  |  |  |  |  |  |  |  |
| No | 334 | 1141.4 | 66 | Ref |  |  |  | Ref |  |  |  |
| Yes | 58 | 166.8 | 6 | 0.5 | 0.18 | 1.13 | 0.125 | 0.58 | 0.21 | 1.38 | 0.253 |
|  |  |  |  |  |  |  |  |  |  |  |  |
| HLA B*57 |  |  |  |  |  |  |  |  |  |  |  |
| No | 360 | 1180.2 | 60 | Ref |  |  |  | Ref |  |  |  |
| Yes | 32 | 128 | 12 | 2.76 | 1.24 | 5.92 | 0.01 | 2.69 | 1.17 | 5.99 | 0.017 |
|  |  |  |  |  |  |  |  |  |  |  |  |
| HLA B*58:02 |  |  |  |  |  |  |  |  |  |  |  |
| No | 336 | 1109.1 | 62 | Ref |  |  |  | Ref |  |  |  |
| Yes | 56 | 199.2 | 10 | 0.93 | 0.42 | 1.88 | 0.841 | 0.82 | 0.36 | 1.74 | 0.619 |
|  |  |  |  |  |  |  |  |  |  |  |  |
| HIV-1 subtype |  |  |  |  |  |  |  |  |  |  |  |
| C | 172 | 525.5 | 24 | Ref |  |  |  | Ref |  |  |  |
| A | 142 | 541.8 | 35 | 1.86 | 1.05 | 3.36 | 0.036 | 1.93 | 1.03 | 3.69 | 0.042 |
| D | 58 | 183.5 | 12 | 1.62 | 0.73 | 3.46 | 0.223 | 1.58 | 0.68 | 3.51 | 0.271 |
| Other | 20 | 57.4 | 1 | 0.34 | 0.02 | 1.78 | 0.305 | 0.35 | 0.02 | 1.91 | 0.326 |
|  |  |  |  |  |  |  |  |  |  |  |  |
| Month 3 CD4 count* | 392 | 1308.2 | 72 | 1.19 | 1.08 | 1.32 | <0.001 | 1.15 | 1.04 | 1.28 | 0.008 |
|  |  |  |  |  |  |  |  |  |  |  |  |
| Total N = 392 volunteers with viral load, subtype, HLA, and baseline CD4 data, including 72 controllers (con)  PY: person years of ART-free follow-up  MSM: Men who have sex with men  * Odds ratio represents the increase in odds of viral control for every 100-cell increase in the month 3 CD4 T cell count | | | | | | | | | | | |

Sensitivity analysis 6: Adopting a more conservative threshold to define control of ≤ 2,000 viral copies/mL we observed only 30 controllers, 25 viremic and 5 aviremic controllers. Our results were similar to those of the main analysis, with females and HLA B*57 remaining associated with control, and the adjusted OR comparing subtype A to subtype C being 3.3 [1.4-8.7] with p = 0.01 (Supplemental Table 6).

| **Supplemental Table 6. Association between baseline covariates and a more conservative definition of viremic control (VL ≤ 2,000) in an early infected cohort (n = 590)** | | | | | | | | | | | |
| --- | --- | --- | --- | --- | --- | --- | --- | --- | --- | --- | --- |
|  |  |  |  | Unadjusted Analysis | | | | Adjusted Analysis | | | |
|  | Control | | |  | 95% CI | |  |  | 95% CI | |  |
|  | n | PY | f | Est OR | LL | UL | p-value | Est OR | LL | UL | p-value |
| Year of EDI |  |  |  |  |  |  |  |  |  |  |  |
| 2005 - 2006 | 170 | 760.5 | 11 | Ref |  |  |  |  |  |  |  |
| 2007 - 2008 | 218 | 812.4 | 10 | 0.76 | 0.31 | 1.84 | 0.538 |  |  |  |  |
| 2009 - 2011 | 202 | 570.2 | 9 | 0.83 | 0.32 | 2.05 | 0.680 |  |  |  |  |
|  |  |  |  |  |  |  |  |  |  |  |  |
| Age at EDI |  |  |  |  |  |  |  |  |  |  |  |
| < 25 years | 160 | 544.8 | 9 | Ref |  |  |  | Ref |  |  |  |
| ≥ 25 years | 430 | 1598.4 | 21 | 0.82 | 0.38 | 1.92 | 0.623 | 1.00 | 0.44 | 2.42 | 0.994 |
|  |  |  |  |  |  |  |  |  |  |  |  |
| Sex |  |  |  |  |  |  |  |  |  |  |  |
| Male | 351 | 1270.9 | 12 | Ref |  |  |  | Ref |  |  |  |
| Female | 239 | 872.4 | 18 | 2.30 | 1.09 | 4.99 | 0.030 | 2.48 | 1.09 | 5.81 | 0.032 |
|  |  |  |  |  |  |  |  |  |  |  |  |
| Risk Group |  |  |  |  |  |  |  |  |  |  |  |
| DC | 427 | 1522.8 | 21 | Ref |  |  |  |  |  |  |  |
| MSM | 90 | 311.7 | 2 | 0.45 | 0.07 | 1.56 | 0.282 |  |  |  |  |
| Other Heterosexual | 65 | 268 | 7 | 2.2 | 0.83 | 5.21 | 0.086 |  |  |  |  |
| Unknown |  |  |  |  |  |  |  |  |  |  |  |
|  |  |  |  |  |  |  |  |  |  |  |  |
| HLA A*02:02 |  |  |  |  |  |  |  |  |  |  |  |
| No | 535 | 1946.9 | 28 | Ref |  |  |  |  |  |  |  |
| Yes | 55 | 196.3 | 2 | 0.69 | 0.11 | 2.41 | 0.624 |  |  |  |  |
|  |  |  |  |  |  |  |  |  |  |  |  |
| HLA A*03 by Sex |  |  |  |  |  |  |  |  |  |  |  |
| Female - No | 213 | 754.1 | 13 | Ref |  |  |  |  |  |  |  |
| Female -Yes | 26 | 118.3 | 5 | 3.29 | 0.97 | 9.78 | 0.039 |  |  |  |  |
| Male - No | 325 | 1166.7 | 11 | 0.54 | 0.23 | 1.22 | 0.138 |  |  |  |  |
| Male - Yes | 26 | 104.2 | 1 | 0.58 | 0.03 | 3.13 | 0.608 |  |  |  |  |
|  |  |  |  |  |  |  |  |  |  |  |  |
| HLA B*45:01 |  |  |  |  |  |  |  |  |  |  |  |
| No | 499 | 1857.8 | 27 | Ref |  |  |  |  |  |  |  |
| Yes | 91 | 285.5 | 3 | 0.64 | 0.15 | 1.87 | 0.474 |  |  |  |  |
|  |  |  |  |  |  |  |  |  |  |  |  |
| HLA B*57 |  |  |  |  |  |  |  |  |  |  |  |
| No | 535 | 1901.6 | 23 | Ref |  |  |  | Ref |  |  |  |
| Yes | 55 | 241.6 | 7 | 2.96 | 1.12 | 6.97 | 0.018 | 3.42 | 1.26 | 8.43 | 0.01 |
|  |  |  |  |  |  |  |  |  |  |  |  |
| HLA B*58:02 |  |  |  |  |  |  |  |  |  |  |  |
| No | 504 | 1819.2 | 25 | Ref |  |  |  |  |  |  |  |
| Yes | 86 | 324 | 5 | 1.16 | 0.38 | 2.9 | 0.766 |  |  |  |  |
|  |  |  |  |  |  |  |  |  |  |  |  |
| HIV-1 subtype |  |  |  |  |  |  |  |  |  |  |  |
| C | 273 | 929.7 | 8 | Ref |  |  |  | Ref |  |  |  |
| A | 207 | 832.1 | 18 | 2.98 | 1.3 | 7.39 | 0.012 | 3.31 | 1.37 | 8.65 | 0.010 |
| D | 81 | 278.4 | 4 | 1.74 | 0.45 | 5.71 | 0.375 | 1.75 | 0.44 | 5.92 | 0.384 |
| other | 29 | 102.9 | 0 | 0.00 | NA | NA | 0.990 | 0.00 | NA | NA | 0.990 |
|  |  |  |  |  |  |  |  |  |  |  |  |
| Month 3 CD4 Count* | 590 | 2143.2 | 30 | 1.19 | 1.05 | 1.33 | 0.003 | 1.14 | 0.99 | 1.29 | 0.050 |
|  |  |  |  |  |  |  |  |  |  |  |  |
| Total N = 590 volunteers with viral load, subtype, and HLA data; PY = person years of ART-free follow-up  * Odds ratio represents the increase in odds of viral control for every 100-cell increase in the month 3 CD4 T cell count | | | | | | | | | | | |

**2. Methods and additional details on the detection of HIV antiretroviral (ARV) therapy drugs used to detect volunteers with low viral loads who had not reported ARV**

Plasma from participants who did not report ART use was tested for the presence of anti-retroviral drugs at the time their viral load dropped to ≤2,000 copies/mL. We also tested plasma retrospectively when an analysis of an individual’s VL trajectory showed a substantial drop (≥2 logs) in the absence of reported ART. Sample was tested for the presence of selected ARV drugs (3TC, D4T, DRV, RTV, LPV, EFV, TFV, FTC, and NVP) at the laboratory of Dr. Edward Acosta at the University of Alabama, Birmingham, US. Sample preparation for protease inhibitors involved addition of an internal standard (A86093·0) and liquid-liquid extraction with 2mL *tert*-butylmethylether (*t*BME) at basic pH, and reconstitution in 100 µL of mobile phase to concentrate the sample. Reversed phase chromatographic separation of the drugs and internal standard was performed on a YMC, C8 analytical column (100 x 4·6mm, 3µm) under isocratic conditions. A binary mobile phase was used consisting of 55% 20 mM sodium acetate buffer (pH 4·88) and 45% acetonitrile. The UV detector set to monitor the 212 nm wavelength provided adequate sensitivity with minimal interference from endogenous matrix components. The calibration curves were linear for all drugs over the range of 50 to 20,000 ng/mL. The inter- and intraday variability at the low and high end of the curve were less than 10%. Sample preparation for nucleoside analogs involved the addition of an internal standard (2’,3’-dideoxyuridine) and solid phase extraction using a Waters OASIS® HLB extraction cartridge 30 mg, 1 cc, and reconstitution in 200 µL of Milli-Q water to concentrate the sample. Reversed phase separation of the drugs and internal standard was performed on a YMC, C8 analytical column (100 x 4.6mm, 3µm) under gradient conditions. The mobile phase consisted of a gradient mixture ranging from 1·3%-85·0% acetonitrile and 98·7%-15·0% 20 mM ammonium formate buffer (pH 3·8). The UV detector set to monitor the 266 nm wavelength provided adequate sensitivity with minimal interference from endogenous matrix components. The calibration curves were linear for all drugs over the range of 25 to 10,000 ng/mL. Sample preparation for tenofovir (TNF) and FTC involved the addition of individual labeled isotopes and a solid phase extraction using a Waters OASIS® MCX extraction cartridge 30 mg, 1 cc, and reconstitution in 100 µL of Milli-Q water to concentrate the sample. Reversed phase chromatographic separation of the drugs and internal standard was performed on a Waters Atlantis, Cd18 analytical column (100 x 2·1mm, 3µm) under isocratic conditions. A binary mobile phase was used consisting of 90 % 0·01% trifluoroacetic acid and 10% acetonitrile. The detection and quantitation was achieved for each drug and internal standard by multiple reaction monitoring (MRM). The protonated molecular ions [M+H]^+^ are monitored at m/z 288·2 > 176·1, 293·2 > 181·0, 248·2 > 130·0, 251·1 > 132·9 for TFV, TFV-IS, FTC, and FTC-IS provided adequate sensitivity with minimal interference from endogenous matrix components. The calibration curves were linear for all drugs over the range of 5 to 2,500 ng/mL. A reversed-phase high performance liquid chromatographic (HPLC) assay, coupled to triple quadrupole mass spectrometer (MS/MS) for detection, was developed and validated for the determination of NVP in human plasma. A simple protein precipitation procedure is performed on the samples using acetonitrile (AcN) which contained stable isotope internal standard (d5-NVP). An aliquot of supernatant is then diluted in 0.1% formic acid before injection onto the HPLC system. Chromatographic separation was performed on a Waters X-Bridge C18, 2.1 x 50 mm, 3·5 μm particle size, analytical column. The mobile phase consisted of 0·1% formic acid in water and 0.1% formic acid in AcN, run isocratically at a ratio of 72:28 % (by volume), at a flow rate of 0·3 mL/min. Detection and quantitation is achieved by multi-reaction monitoring (MRM) of protonated products [M+H]^+^ using the following transitions: NVP - *m/z* 267·2>107·1 and NVP-IS - m/z 272·3>228·1. The assay was linear in the range of 5-5000 ng/mL using a 20 μL aliquot of human plasma. Results are shown below in ng/mL (Supplemental Table 6). The limit of detection for each drug screened was as follows: 3TC 25 ng/mL, AZT 25 ng/mL, D4T 25 ng/mL, DRV 50 ng/mL, RTV 50 ng/mL, LPV 50 ng/mL, EFV 50 ng/mL, TFV 5 ng/mL, FTC 5 ng/mL, and NVP 5 ng/mL. Results shown as zero (0) should be interpreted as “below the detection threshold of the assay”. Results shown as blank represent an assay that was not done, or an assay failure (see comments column). Testing was done at the laboratories of Dr. E Acosta at University of Alabama, Birmingham, AL, USA (1) and Dr. R Schinazi at Emory University, Atlanta, GA, USA (2).

**Supplementary Table 6: Results of ARV drug detection assays:**

| id | visitcode | visitdate | Any ARV detected? | 3tc ng/mL | azt ng/mL | ftc ng/mL | tfv ng/mL | d4t ng/mL | efv ng/mL | lpv ng/mL | rtv ng/mL | nvp ng/mL | drv ng/mL | ddI ng/mL | atv ng/mL | abc ng/mL | Lab | Comments |
| --- | --- | --- | --- | --- | --- | --- | --- | --- | --- | --- | --- | --- | --- | --- | --- | --- | --- | --- |
| 1 | 0.1 | 22-Feb-06 | No |  |  | 0 | 0 |  | 0 | 0 | 0 | 0 | 0 |  |  |  | (1) |  |
| 2 | 90.0 | 8-Jun-12 | No | 0 | 0 | 0 | 0 | 0 | 0 | 0 | 0 | 0 | 0 |  |  |  | (2) |  |
| 3 |  | 3-Mar-06 | No | 0 | 0 | 0 | 0 | 0 | 0 | 0 | 0 | 0 | 0 |  |  |  | (1) | 1 |
| 4 | 0.1 | 8-Mar-06 | No |  |  | 0 | 0 |  | 0 | 0 | 0 | 0 | 0 |  |  |  | (1) |  |
| 5 | 6.0 | 31-May-06 | No |  |  | 0 | 0 |  | 0 | 0 | 0 | 0 | 0 |  |  |  | (1) |  |
| 6 | 21.0 | 23-Nov-06 | No | 0 | 0 | 0 | 0 | 0 | 0 | 0 | 0 | 0 | 0 | 0 | 0 | 0 | (2) |  |
| 7 | 36.0 | 4-Sep-08 | Yes |  |  | 0 | 0 |  | 0 | 0 | 0 | 101 | 0 |  |  |  | (1) |  |
| 8 | 12.0 | 24-Apr-07 | No |  |  | 0 | 0 |  | 0 | 0 | 0 | 0 | 0 |  |  |  | (1) |  |
| 9 | 0.1 | 7-Oct-06 | No |  |  | 0 | 0 |  | 0 | 0 | 0 | 0 | 0 |  |  |  | (1) |  |
| 10 | 15.0 | 11-Jan-08 | No |  |  | 0 | 0 |  | 0 | 0 | 0 | 0 | 0 |  |  |  | (1) |  |
| 11 | 6.0 | 8-Jun-07 | No |  |  | 0 | 0 |  | 0 | 0 | 0 | 0 | 0 |  |  |  | (1) |  |
| 12 | 3.0 | 18-Jun-07 | No |  |  | 0 | 0 |  | 0 | 0 | 0 | 0 | 0 |  |  |  | (1) |  |
| 12 | 30.0 | 7-Jul-09 | No | 0 | 0 | 0 | 0 | 0 | 0 | 0 | 0 | 0 | 0 |  |  |  | (1) |  |
| 13 | 66.0 | 10-Aug-12 | No | 0 | 0 | 0 | 0 | 0 | 0 | 0 | 0 | 0 | 0 |  |  |  | (2) |  |
| 14 | 3.0 | 21-Apr-08 | No |  |  | 0 | 0 |  | 0 | 0 | 0 | 0 | 0 |  |  |  | (1) |  |
| 15 | 1.0 | 2-Jul-08 | No | 0 | 0 | 0 | 0 | 0 | 0 | 0 | 0 | 0 | 0 |  |  |  | (2) |  |
| 16 | 6.0 | 18-Dec-08 | No |  |  | 0 | 0 |  | 0 | 0 | 0 | 0 | 0 |  |  |  | (1) |  |
| 17 | 95.1 | 24-Dec-09 | No | 0 | 0 | 0 | 0 | 0 | 0 | 0 | 0 | 0 | 0 |  |  |  | (2) |  |
| 18 | 36.0 | 11-Jun-08 | Yes | 0 | 0 | 0 | 0 | 0 | 0 | 0 | 36.9 | 0 | 0 |  |  |  | (2) |  |
| 19 | 12.0 | 5-Feb-07 | No |  |  | 0 | 0 |  | 0 | 0 | 0 | 0 | 0 |  |  |  | (1) |  |
| 20 | 12.0 | 23-Mar-09 | No |  |  | 0 | 0 |  | 0 | 0 | 0 | 0 | 0 |  |  |  | (1) |  |
| 21 | 9.0 | 13-Nov-07 | No | 0 | 0 | 0 | 0 | 0 | 0 | 0 | 0 | 0 | 0 | 0 | 0 | 0 | (2) |  |
| 21 | 48.0 | 12-Nov-10 | No | 0 | 0 | 0 | 0 | 0 | 0 | 0 | 0 | 0 | 0 |  |  |  | (1) |  |
| 22 | 6.0 | 27-Apr-07 | No |  |  | 0 | 0 |  | 0 | 0 | 0 | 0 | 0 |  |  |  | (1) |  |
| 23 | 42.0 | 14-Jun-10 | Yes | 0 | 0 | 0 | 0 | 0 | 0 | 0 | 0 | 0.6 | 0 | 0 | 0 | 0 | (2) |  |
| 24 | 15.0 | 22-May-09 | No | 0 | 0 | 0 | 0 | 0 | 0 | 0 | 0 | 0 | 0 |  |  |  | (2) |  |
| 24 | 18.0 | 17-Aug-09 | Yes |  |  | 0 | 0 |  | 0 | 0 | 0 | 678.5 | 835 |  |  |  | (1) |  |
| 25 | 2.0 | 27-Apr-07 | No |  |  | 0 | 0 |  | 0 | 0 | 0 | 0 | 0 |  |  |  | (1) |  |
| 26 | 0.1 | 4-Oct-07 | No |  |  | 0 | 0 |  | 0 | 0 | 0 | 0 | 0 |  |  |  | (1) |  |
| 27 | 0.1 | 29-Aug-08 | No |  |  | 0 | 0 |  | 0 | 0 | 0 | 0 | 0 |  |  |  | (1) |  |
| 28 | 0.1 | 19-Sep-08 | Yes |  |  | 0 | 0 |  | 0 | 0 | 0 | 1318.9 | 0 |  |  |  | (1) |  |
| 29 | 12.0 | 14-Oct-08 | Yes | 0 | 0 | 0 | 0 | 0 | 0 | 0 | 0 | 1.04 | 0 | 0 | 0 | 0 | (2) |  |
| 29 | 42.0 | 7-Feb-11 | No | 0 | 0 | 0 | 0 | 0 | 0 | 0 | 0 | 0 | 0 |  |  |  | (1) |  |
| 30 | 42.0 | 23-Aug-10 | No | 0 | 0 | 0 | 0 | 0 | 0 | 0 | 0 | 0 | 0 |  |  |  | (1) |  |
| 31 | 3.0 | 29-Jan-09 | No |  |  | 0 | 0 |  | 0 | 0 | 0 | 0 | 0 |  |  |  | (1) |  |
| 32 | 3.0 | 18-Aug-08 | No |  |  | 0 | 0 |  | 0 | 0 | 0 | 0 | 0 |  |  |  | (1) |  |
| 33 | 2.0 | 27-Nov-07 | No | 0 | 0 | 0 | 0 | 0 | 0 | 0 | 0 | 0 | 0 |  |  |  | (2) |  |
| 33 | 15.0 | 24-Nov-08 | Yes | 0 | 0 | 0 | 0 | 0 | 0 | 0 | 0 | 1.2 | 0 |  |  |  | (2) |  |
| 34 | 6.0 | 30-Nov-09 | Yes |  |  | 0 | 0 |  | 0 | 0 | 0 | 1150.3 | 0 |  |  |  | (1) |  |
| 35 | 3.0 | 1-Nov-10 | No | 0 | 0 | 0 | 0 | 0 | 0 | 0 | 0 | 0 | 0 |  |  |  | (1) |  |
| 36 | 3.0 | 5-Jan-07 | No |  |  | 0 | 0 |  | 0 | 0 | 0 | 0 | 0 |  |  |  | (1) |  |
| 37 | 0.1 | 29-Nov-06 | No |  |  | 0 | 0 |  |  |  |  | 0 |  |  |  |  | (1) | 2 |
| 38 | 0.1 | 5-Sep-07 | No | 0 | 0 | 0 | 0 | 0 | 0 | 0 | 0 | 0 | 0 |  |  |  | (1) |  |
| 39 | 21.0 | 25-Jun-09 | No | 0 | 0 | 0 | 0 | 0 | 0 | 0 | 0 | 0 | 0 |  |  |  | (1) |  |
| 40 | 6.0 | 20-Jun-08 | No |  |  | 0 | 0 |  | 0 | 0 | 0 | 0 | 0 |  |  |  | (1) |  |
| 41 | 12.0 | 12-Feb-09 | No |  |  | 0 | 0 |  | 0 | 0 | 0 | 0 | 0 |  |  |  | (1) |  |
| 42 | 0.1 | 29-Jun-09 | No |  |  | 0 | 0 |  | 0 | 0 | 0 | 0 | 0 |  |  |  | (1) |  |
| 43 | 2.0 | 2-Dec-09 | No |  |  | 0 | 0 |  | 0 | 0 | 0 | 0 | 0 |  |  |  | (1) |  |
| 44 | 21.0 | 21-Oct-11 | Yes | 66.4 | 684 | 0 | 0 | 0 | 1,219 | 0 | 0 | 0 | 0 | 0 | 0 | 0 | (2) |  |
| 45 | 95.5 | 27-Oct-10 | Yes | 0 | 79.3 | 0 | 35.2 | 0 | 0 | 0 | 0 | 0 | 62.4 |  |  |  | (1) |  |
| 46 | 3.0 | 10-Dec-10 | No | 0 | 0 | 0 | 0 | 0 | 0 | 0 | 0 | 0 | 0 |  |  |  | (1) |  |
| 47 | 12.0 | 20-Jan-12 | No | 0 | 0 | 0 | 0 | 0 | 0 | 0 | 0 | 0 | 0 |  |  |  | (2) |  |
| 48 | 9.0 | 2-May-12 | Yes | 70.8 | 0 | 0 | 61.4 | 0 | 3,632 | 0 | 0 | 0 | 0 | 0 | 0 | 0 | (2) |  |
| 49 | 18.0 | 10-Feb-13 | No | 0 | 0 | 0 | 0 | 0 | 0 | 0 | 0 | 0 | 0 | 0 | 0 | 0 | (2) |  |
| 50 | 36.1 | 5-Sep-11 | No | 0 | 0 | 0 | 0 | 0 | 0 | 0 | 0 | 0 | 0 |  |  |  | (2) |  |
| 51 | 3.0 | 21-Aug-07 | No |  |  | 0 | 0 |  | 0 | 0 | 0 | 0 | 0 |  |  |  | (1) |  |
| 51 | 30.0 | 11-Sep-09 | No | 0 | 0 | 0 | 0 | 0 | 0 | 0 | 0 | 0 | 0 |  |  |  | (1) |  |
| 52 | 9.0 | 31-Oct-07 | No | 0 | 0 | 0 | 0 | 0 | 0 | 0 | 0 | 0 | 0 | 0 | 0 | 0 | (2) |  |
| 52 | 42.0 | 14-May-10 | No | 0 | 0 | 0 | 0 | 0 | 0 | 0 | 0 | 0 | 0 |  |  |  | (1) |  |
| 53 | 9.0 | 27-Jul-09 | No |  |  | 0 | 0 |  | 0 | 0 | 0 | 0 | 0 |  |  |  | (1) |  |
| 54 | 3.0 | 6-Oct-09 | No |  |  | 0 | 0 |  | 0 | 0 | 0 | 0 | 0 |  |  |  | (1) |  |
| 55 | 0.1 | 10-Jul-09 | No | 0 | 0 | 0 | 0 | 0 | 0 | 0 | 0 | 0 | 0 |  |  |  | (1) |  |
| 56 | 18.0 | 19-Jan-11 | No | 0 | 0 | 0 | 0 | 0 | 0 | 0 | 0 | 0 | 0 |  |  |  | (2) |  |
| 56 | 24.0 | 5-Jul-11 | Yes | 0 | 0 | 0 | 0 | 0 | 0 | 0 | 0 | 0 | 150.8 |  |  |  | (1) |  |
| 57 | 18.0 | 18-Oct-11 | Yes | 194.7 | 0 | 0 | 22.2 | 0 | 4293 | 0 | 0 | 0 | 0 |  |  |  | (2) |  |
| 58 | 54.1 | 14-Apr-14 | No | 0 | 0 | 0 | 0 | 0 | 0 | 0 | 0 | 0 | 0 | 0 | 0 | 0 | (2) |  |
| 59 | 12.0 | 10-Oct-06 | Yes | 0 | 0 | 0 | 0 | 0 | 0 | 0 | 0 | 0.32 | 0 | 0 | 0 | 0 | (2) |  |
| 60 | 54.0 | 26-May-10 | No | 0 | 0 | 0 | 0 | 0 | 0 | 0 | 0 | 0 | 0 |  |  |  | (1) |  |
| 61 | 30.0 | 13-May-09 | Yes | 0 | 0 | 11.7 | 58.6 | 0 | 0 | 0 | 0 | 4,038 | 0 | 0 | 0 | 0 | (2) |  |
| 62 | 36.0 | 5-Dec-09 | Yes |  |  | 174.4 | 64.4 |  | 1111.2 | 0 | 0 | 0 | 0 |  |  |  | (1) |  |
| 63 | 3.0 | 22-Jan-08 | No |  |  | 0 | 0 |  | 0 | 0 | 0 | 0 | 0 |  |  |  | (1) |  |
| 64 | 60.0 | 28-Apr-12 | No | 0 | 0 | 0 | 0 | 0 | 0 | 0 | 0 | 0 | 0 |  |  |  | (2) |  |
| 65 | 48.0 | 13-Aug-11 | No | 0 | 0 | 0 | 0 | 0 | 0 | 0 | 0 | 0 | 0 | 0 | 0 | 0 | (2) |  |
| 66 | 6.0 | 4-Sep-08 | No | 0 | 0 | 0 | 0 | 0 | 0 | 0 | 0 | 0 | 0 |  |  |  | (2) |  |
| 67 | 12.0 | 4-Apr-12 | Yes | 0 | 0 | 0 | 0 | 0 | 0 | 0.34 | 0.79 | 2.77 | 0 |  |  |  | (2) |  |
| 68 | 9.0 | 10-Jan-07 | No | 0 | 0 | 0 | 0 | 0 | 0 | 0 | 0 | 0 | 0 | 0 | 0 | 0 | (2) |  |
| 69 | 0.1 | 2-Aug-07 | No |  |  | 0 | 0 |  | 0 | 0 | 0 | 0 | 0 |  |  |  | (1) |  |
| 70 | 21.0 | 6-Apr-09 | Yes | 668.8 | 0 | 0 | 0 | 0 | 0 | 0 | 0 | 6068.3 | 140.1 |  |  |  | (1) |  |
| 70 | 24.0 | 15-Jul-09 | No |  |  | 0 | 0 |  | 0 | 0 | 0 | 0 | 0 |  |  |  | (1) |  |
| 71 | 3.0 | 20-Apr-09 | No |  |  | 0 | 0 |  | 0 | 0 | 0 | 0 | 0 |  |  |  | (1) |  |
| 72 | 0.1 | 9-Jul-09 | No |  |  | 0 | 0 |  | 0 | 0 | 0 | 0 | 0 |  |  |  | (1) |  |
| 73 | 6.0 | 31-May-10 | Yes | 0 | 0 | 0 | 23.9 | 0 | 0 | 0 | 0 | 0 | 56.2 |  |  |  | (1) |  |
| 74 | 0.1 | 7-Apr-10 | No | 0 | 0 | 0 | 0 | 0 | 0 | 0 | 0 | 0 | 0 |  |  |  | (1) |  |
| 75 | 2.0 | 12-Oct-10 | No | 0 | 0 | 0 | 0 | 0 | 0 | 0 | 0 | 0 | 1* |  |  |  | (1) | 3 |
| 76 | 9.0 | 24-Aug-07 | Yes | 0 | 0 | 0 | 0 | 0 | 0 | 0 | 0 | 1.2 | 0 |  |  |  | (2) |  |
| 76 | 24.0 | 10-Oct-08 | No | 0 | 0 | 0 | 0 | 0 | 0 | 0 | 0 | 0 | 0 |  |  |  | (2) |  |
| 77 | 9.0 | 16-Feb-07 | No | 0 | 0 | 0 | 0 | 0 | 0 | 0 | 0 | 0 | 0 | 0 | 0 | 0 | (2) |  |
| 78 | 36.0 | 17-Dec-08 | No |  |  | 0 | 0 |  | 0 | 0 | 0 | 0 | 0 |  |  |  | (1) |  |
| 79 | 36.0 | 16-Jun-10 | Yes | 0 | 0 | 2.72 | 87.7 | 0 | 2,193 | 0 | 0 | 0 | 0 | 0 | 0 | 0 | (2) |  |
| 80 | 3.0 | 19-Jun-09 | No |  |  | 0 | 0 |  | 0 | 0 | 0 | 0 | 0 |  |  |  | (1) |  |
| 81 | 9.0 | 7-Apr-10 | No | 0 | 0 | 0 | 0 | 0 | 0 | 0 | 0 | 0 | 0 | 0 | 0 | 0 | (2) |  |
| 82 | 2.0 | 25-Jun-10 | No | 0 | 0 | 0 | 0 | 0 | 0 | 0 | 0 | 0 | 0 |  |  |  | (1) |  |
| 83 | 12.0 | 13-Apr-11 | No | 0 | 0 | 0 | 0 | 0 | 0 | 0 | 0 | 0 | 0 |  |  |  | (1) |  |
| 84 | 95.1 | 30-Jun-10 | No | 0 | 0 | 0 | 0 | 0 | 0 | 0 | 0 | 0 | 0 |  |  |  | (2) |  |
| 84 | 12.1 | 3-Jun-11 | Yes | 0 | 0 | 0 | 0 | 0 | 0 | 0 | 0 | 2.17 | 0 | 0 | 0 | 0 | (2) |  |
| 85 | 2.0 | 10-Mar-11 | No | 0 | 0 | 0 | 0 | 0 | 0 | 0 | 0 | 0 | 0 |  |  |  | (1) |  |
| 86 | 3.0 | 11-Oct-11 | No | 0 | 0 | 0 | 0 | 0 | 0 | 0 | 0 | 0 | 0 |  |  |  | (2) |  |
| 87 | 15.0 | 24-Jul-12 | Yes | 0 | 0 | 0 | 30.2 | 0 | 0 | 0 | 0 | 0 | 0 |  |  |  | (2) |  |
| 88 | 30.1 | 9-Sep-11 | No | 0 | 0 | 0 | 0 | 0 | 0 | 0 | 0 | 0 | 0 |  |  |  | (2) |  |
| 89 | 3.0 | 19-Jan-10 | No | 0 | 0 | 0 | 0 | 0 | 0 | 0 | 0 | 0 | 0 |  |  |  | (1) |  |
| Visitcode is a unique identifier assigned to each study visit that corresponds approximately with months post estimated date of HIV-1 infection. Visitcode 95.X typically designated visits within one month of EDI. | | | | | | | | | | | | | | | | | | |
| ARV concentrations are shown in nanograms per mililiter where 0 is below assay detection threshold and missing is not done or assay failure (see comments) | | | | | | | | | | | | | | | | | | |
| Note that some volunteers had two samples tested, thus the duplicate id entries  Laboratories:   1. Dr. E Acosta at University of Alabama, Birmingham, AL, USA 2. Dr. R Schinazi at Emory University, Atlanta, GA, USA   Comments:   1. Interim or other type of visit, no study-designated visit code 2. Several failed (missing data) 3. DRV at or below assay detection limits, likely assay interference. Upon consultation with laboratory team, we considered this volunteer to be ARV negative | | | | | | | | | | | | | | | | | | |

**3. Additional details on ethical review**

This study was reviewed and approved by the following ethical review boards: the Kenya Medical Research Institute Ethical Review Committee, the Kenyatta National Hospital Ethical Review Committee of the University of Nairobi, the Rwanda National Ethics Committee, the Uganda Virus Research Institute Science and Ethics Committee (Currently the UVRI Research Ethics Committee) and the Uganda National Council of Science and Technology, the University of Cape Town Health Science Research and Ethics Committee, the Bio-Medical Research Ethics Committee at the University of KwaZulu Natal, the University of Zambia Research Ethics Committee, and the Emory University Institutional Review Board. All volunteers completed an informed consent procedure prior to the collection of any study-related data; demonstrating comprehension on a standardized informed consent assessment of understanding was a requirement for study participation.

1. **Cohort recruitment**

Volunteers were primarily recruited from HIV-1 epidemiology studies at 9 clinical research centers in Kenya, Rwanda, South Africa, Uganda, and Zambia. These cohort studies followed thousands of persons at risk for HIV infection and included monthly or quarterly HIV testing, counseling, and condom provision. For more information, see (Kamali, Price et al. 2015). Risk for HIV-1 infection was defined as regular sexual activity with an HIV infected partner not currently on ART (Zambia, Rwanda, and Uganda), men who report sex with men (Kenya and South Africa), report of transactional sex (Kenya), and report of sexual activity along with another risk factor such as young age (Cape Town) or place of residence - either rural communities with high HIV prevalence (Uganda), fishing communities (Uganda) or peri-urban communities (South Africa). Testing included HIV antibody rapid tests (Determine®, Abbot Laboratories, Japan and Uni-Gold™, Trinity Biotechnology, Ireland), ELISA confirmation where indicated by national guidelines (HIV-1/2 ELISA Vironostika® Uni-Form II Ag/Ab, Biomerieux, The Netherlands; Detect-HIV™ ELISA, Adaltis, Inc., Italy; or Murex HIV-1·2·0 ELISA, Abbott, USA), and HIV p24 antigen testing (Coulter p24 HIV-1 Antigen Assay through March 2007; Vironostika® HIV-1 p24 Antigen thereafter) to detect HIV prior to antibody seroconversion. Where available, the specimen taken immediately prior to the detection of HIV was tested by PCR for the presence of pre-seroconversion HIV (COBAS® Amplicor Monitor v1·5, Roche, Switzerland through January 2011; Abbott Real Time HIV-1 v1·0 m2000sp/m2000rt thereafter).

The estimated date of infection (EDI) was defined as the midpoint between the date of the last negative and first positive test in the case of detection by HIV antibody assay, 14 days prior to the test date in the case of detection by p24 antigen assay only, or 10 days prior to the test date for those volunteers with a PCR-positive result prior to antibody or p24 antigen detection. If a volunteer could identify a clear and obvious exposure event, the date of this event could be adopted as the EDI at the discretion of the research team.

Additional details on imputed data: The 590 volunteers with sufficient data includes 5 volunteers for whom subtype was imputed. Two volunteers from South Africa and without partner subtype data were assumed to be infected with subtype C. Three volunteers with partner data (one each from Rwanda, Uganda, and Zambia) were assumed to be infected with the subtype of their partner, subtype A.

1. **Genbank Accession Numbers**

| volunteer_id | AccessionID |
| --- | --- |
| 95742 | KC018990 |
| 95337 | KC018613 |
| 95378 | KC018652 |
| 95650 | KC018906 |
| 95400 | KC018671 |
| 95523 | KC018786 |
| 95534 | KC018794 |
| 95502 | KC018768 |
| 95820 | KC019059 |
| 95341 | KC018617 |
| 95225 | KC018509 |
| 95571 | KC018827 |
| 95824 | KC019062 |
| 95723 | KC018972 |
| 95258 | KC018540 |
| 95774 | KC019021 |
| 95796 | KC019042 |
| 95707 | KC018956 |
| 95864 | KC019095 |
| 95438 | KC018705 |
| 95880 | KC019105 |
| 95233 | KC018517 |
| 95376 | KC018650 |
| 95386 | KC018659 |
| 95471 | KC018736 |
| 95786 | KC019031 |
| 95352 | KC018627 |
| 95670 | KC018923 |
| 95860 | KC019091 |
| 95889 | KC019114 |
| 95261 | KC018543 |
| 95854 | KC019086 |
| 95858 | KC019090 |
| 95375 | KC018649 |
| 95499 | KC018766 |
| 95758 | KC019005 |
| 95447 | KC018714 |
| 95493 | KC018760 |
| 95239 | KC018524 |
| 95204 | KC018482 |
| 95780 | KC019026 |
| 95821 | KC019060 |
| 95473 | KC018739 |
| 95521 | KC018784 |
| 95532 | KC018792 |
| 95641 | KC018896 |
| 95597 | KC018857 |
| 95463 | KC018728 |
| 95736 | KC018984 |
| 95518 | KC018781 |
| 95448 | KC018715 |
| 95561 | KC018814 |
| 95328 | KC018604 |
| 95883 | KC019108 |
| 95853 | KC019085 |
| 95389 | KC018661 |
| 95509 | KC018774 |
| 95607 | KC018867 |
| 95301 | KC018579 |
| 95606 | KC018866 |
| 95735 | KC018983 |
| 95365 | KC018639 |
| 95862 | KC019093 |
| 95556 | KC018810 |
| 95691 | KC018941 |
| 95264 | KC018546 |
| 95885 | KC019110 |
| 95452 | KC018718 |
| 95202 | KC018479 |
| 95475 | KC018741 |
| 95887 | KC019112 |
| 95353 | KC018628 |
| 95567 | KC018821 |
| 95374 | KC018648 |
| 95828 | KC019065 |
| 95573 | KC018830 |
| 95362 | KC018636 |
| 95793 | KC019038 |
| 95459 | KC018724 |
| 95613 | KC018871 |
| 95349 | KC018625 |
| 95773 | KC019020 |
| 95217 | KC018500 |
| 95686 | KC018937 |
| 95622 | KC018879 |
| 95334 | KC018610 |
| 95560 | KC018813 |
| 95646 | KC018902 |
| 95298 | KC018576 |
| 95254 | KC018537 |
| 95355 | KC018630 |
| 95843 | KC019077 |
| 95693 | KC018944 |
| 95316 | KC018595 |
| 95469 | KC018734 |
| 95694 | KC018945 |
| 95402 | KC018673 |
| 95361 | KC018635 |
| 95442 | KC018709 |
| 95726 | KC018975 |
| 95536 | KC018796 |
| 95401 | KC018672 |
| 95717 | KC018967 |
| 95209 | KC018488 |
| 95590 | KC018850 |
| 95426 | KC018694 |
| 95297 | KC018575 |
| 95740 | KC018989 |
| 95383 | KC018656 |
| 95618 | KC018876 |
| 95857 | KC019089 |
| 95651 | KC018907 |
| 95867 | KC019098 |
| 95783 | KC019029 |
| 95769 | KC019015 |
| 95794 | KC019039 |
| 95812 | KC019053 |
| 95510 | KC018775 |
| 95863 | KC019094 |
| 95861 | KC019092 |
| 95772 | KC019019 |
| 95623 | KC018880 |
| 95419 | KC018687 |
| 95392 | KC018665 |
| 95294 | KC018572 |
| 95591 | KC018851 |
| 95330 | KC018606 |
| 95779 | KC019025 |
| 95595 | KC018855 |
| 95751 | KC018999 |
| 95411 | KC018680 |
| 95490 | KC018759 |
| 95816 | KC019057 |
| 95236 | KC018520 |
| 95422 | KC018690 |
| 95371 | KC018644 |
| 95546 | KC018804 |
| 95844 | KC019078 |
| 95810 | KC019051 |
| 95228 | KC018512 |
| 95454 | KC018720 |
| 95405 | KC018676 |
| 95782 | KC019028 |
| 95617 | KC018875 |
| 95676 | KC018929 |
| 95813 | KC019054 |
| 95231 | KC018515 |
| 95302 | KC018580 |
| 95407 | KC018677 |
| 95850 | KC019082 |
| 95213 | KC018494 |
| 95494 | KC018761 |
| 95725 | KC018974 |
| 95278 | KC018560 |
| 95273 | KC018557 |
| 95871 | KC019100 |
| 95530 | KC018790 |
| 95434 | KC018702 |
| 95290 | KC018570 |
| 95388 | KC018660 |
| 95621 | KC018878 |
| 95272 | KC018556 |
| 95465 | KC018731 |
| 95432 | KC018700 |
| 95381 | KC018654 |
| 95672 | KC018925 |
| 95201 | KC018478 |
| 95638 | KC018893 |
| 95445 | KC018712 |
| 95734 | KC018981 |
| 95508 | KC018773 |
| 95506 | KC018771 |
| 95596 | KC018856 |
| 95807 | KC019049 |
| 95538 | KC018798 |
| 95474 | KC018740 |
| 95485 | KC018754 |
| 95659 | KC018915 |
| 95450 | KC018717 |
| 95326 | KC018603 |
| 95764 | KC019010 |
| 95227 | KC018511 |
| 95829 | KC019066 |
| 95681 | KC018933 |
| 95877 | KC019104 |
| 95647 | KC018903 |
| 95731 | KC018979 |
| 95329 | KC018605 |
| 95865 | KC019096 |
| 95710 | KC018959 |
| 95558 | KC018811 |
| 95811 | KC019052 |
| 95666 | KC018920 |
| 95274 | KC018558 |
| 95831 | KC019067 |
| 95673 | KC018926 |
| 95776 | KC019022 |
| 95866 | KC019097 |
| 95874 | KC019103 |
| 95286 | KC018567 |
| 95683 | KC018934 |
| 95747 | KC018995 |
| 95318 | KC018597 |
| 95842 | KC019076 |
| 95559 | KC018812 |
| 95572 | KC018828 |
| 95367 | KC018640 |
| 95480 | KC018747 |
| 95440 | KC018707 |
| 95770 | KC019016 |
| 95569 | KC018824 |
| 95466 | KC018732 |
| 95851 | KC019083 |
| 95716 | KC018966 |
| 95718 | KC018968 |
| 95712 | KC018961 |
| 95663 | KC018919 |
| 95704 | KC018953 |
| 95232 | KC018516 |
| 95321 | KC018599 |
| 95528 | KC018789 |
| 95300 | KC018578 |
| 95253 | KC018536 |
| 95697 | KC018948 |
| 95260 | KC018542 |
| 95756 | KC019003 |
| 95701 | KC018951 |
| 95753 | KC019000 |
| 95223 | KC018508 |
| 95309 | KC018587 |
| 95642 | KC018897 |
| 95277 | KC018559 |
| 95610 | KC018868 |
| 95291 | KC018571 |
| 95699 | KC018950 |
| 95592 | KC018852 |
| 95719 | KC018969 |
| 95420 | KC018688 |
| 95585 | KC018845 |
| 95404 | KC018675 |
| 95627 | KC018885 |
| 95343 | KC018620 |
| 95798 | KC019043 |
| 95240 | KC018525 |
| 95870 | KC019099 |
| 95648 | KC018904 |
| 95755 | KC019002 |
| 95344 | KC018621 |
| 95669 | KC018922 |
| 95501 | KC018767 |
| 95235 | KC018519 |
| 95259 | KC018541 |
| 95377 | KC018651 |
| 95431 | KC018699 |
| 95372 | KC018645 |
| 95601 | KC018860 |
| 95656 | KC018913 |
| 95357 | KC018631 |
| 95520 | KC018783 |
| 95481 | KC018748 |
| 95667 | KC018921 |
| 95655 | KC018912 |
| 95305 | KC018584 |
| 95347 | KC018624 |
| 95593 | KC018853 |
| 95461 | KC018726 |
| 95482 | KC018749 |
| 95729 | KC018977 |
| 95881 | KC019106 |
| 95708 | KC018957 |
| 95458 | KC018723 |
| 95262 | KC018544 |
| 95713 | KC018962 |
| 95713 | KC018963 |
| 95687 | KC018938 |
| 95444 | KC018711 |
| 95705 | KC018954 |
| 95360 | KC018634 |
| 95269 | KC018553 |
| 95688 | KC018939 |
| 95743 | KC018991 |
| 95838 | KC019072 |
| 95222 | KC018507 |
| 95410 | KC018679 |
| 95391 | KC018664 |
| 95247 | KC018530 |
| 95649 | KC018905 |
| 95702 | KC018952 |
| 95662 | KC018918 |
| 95256 | KC018538 |
| 95295 | KC018573 |
| 95385 | KC018658 |
| 95369 | KC018642 |
| 95582 | KC018842 |
| 95504 | KC018770 |
| 95739 | KC018987 |
| 95580 | KC018839 |
| 95342 | KC018618 |
| 95418 | KC018686 |
| 95692 | KC018942 |
| 95675 | KC018928 |
| 95615 | KC018873 |
| 95873 | KC019102 |
| 95570 | KC018825 |
| 95477 | KC018743 |
| 95271 | KC018555 |
| 95680 | KC018932 |
| 95562 | KC018815 |
| 95626 | KC018883 |
| 95584 | KC018844 |
| 95441 | KC018708 |
| 95439 | KC018706 |
| 95252 | KC018535 |
| 95767 | KC019013 |
| 95484 | KC018752 |
| 95586 | KC018846 |
| 95211 | KC018490 |
| 95453 | KC018719 |
| 95296 | KC018574 |
| 95390 | KC018662 |
| 95605 | KC018865 |
| 95855 | KC019087 |
| 95398 | KC018669 |
| 95795 | KC019040 |
| 95395 | KC018667 |
| 95594 | KC018854 |
| 95415 | KC018685 |
| 95568 | KC018822 |
| 95313 | KC018592 |
| 95203 | KC018480 |
| 95205 | KC018484 |
| 95424 | KC018692 |
| 95577 | KC018834 |
| 95234 | KC018518 |
| 95373 | KC018646 |
| 95464 | KC018729 |
| 95511 | KC018776 |
| 95765 | KC019011 |
| 95221 | KC018505 |
| 95304 | KC018582 |
| 95771 | KC019017 |
| 95212 | KC018492 |
| 95216 | KC018498 |
| 95220 | KC018503 |
| 95624 | KC018881 |
| 95472 | KC018737 |
| 95749 | KC018997 |
| 95746 | KC018993 |
| 95268 | KC018551 |
| 95282 | KC018563 |
| 95266 | KC018548 |
| 95430 | KC018697 |
| 95449 | KC018716 |
| 95778 | KC019023 |
| 95836 | KC019071 |
| 95787 | KC019032 |
| 95825 | KC019063 |
| 95565 | KC018819 |
| 95578 | KC018836 |
| 95714 | KC018964 |
| 95539 | KC018799 |
| 95206 | KC018485 |
| 95340 | KC018616 |
| 95583 | KC018843 |
| 95237 | KC018521 |
| 95760 | KC019007 |
| 95882 | KC019107 |
| 95674 | KC018927 |
| 95229 | KC018513 |
| 95306 | KC018585 |
| 95652 | KC018908 |
| 95587 | KC018848 |
| 95553 | KC018809 |
| 95525 | KC018787 |
| 95368 | KC018641 |
| 95724 | KC018973 |
| 95413 | KC018683 |
| 95479 | KC018746 |
| 95496 | KC018763 |
| 95242 | KC018527 |
| 95625 | KC018882 |
| 95818 | KC019058 |
| 95215 | KC018497 |
| 95325 | KC018602 |
| 95487 | KC018756 |
| 95470 | KC018735 |
| 95750 | KC018998 |
| 95317 | KC018596 |
| 95846 | KC019080 |
| 95645 | KC018901 |
| 95384 | KC018657 |
| 95792 | KC019037 |
| 95732 | KC018980 |
| 95517 | KC018780 |
| 95640 | KC018895 |
| 95537 | KC018797 |
| 95834 | KC019069 |
| 95754 | KC019001 |
| 95588 | KC018849 |
| 95311 | KC018590 |
| 95324 | KC018601 |
| 95540 | KC018800 |
| 95478 | KC018745 |
| 95283 | KC018565 |
| 95766 | KC019012 |
| 95315 | KC018594 |
| 95403 | KC018674 |
| 95803 | KC019047 |
| 95612 | KC018870 |
| 95263 | KC018545 |
| 95456 | KC018721 |
| 95332 | KC018608 |
| 95257 | KC018539 |
| 95579 | KC018838 |
| 95214 | KC018496 |
| 95549 | KC018807 |
| 95219 | KC018502 |
| 95745 | KC018992 |
| 95345 | KC018622 |
| 95346 | KC018623 |
| 95359 | KC018633 |
| 95358 | KC018632 |
| 95634 | KC018889 |
| 95614 | KC018872 |
| 95748 | KC018996 |
| 95602 | KC018862 |
| 95429 | KC018696 |
| 95581 | KC018841 |
| 95489 | KC018757 |
| 95886 | KC019111 |
| 95428 | KC018695 |
| 95370 | KC018643 |
| 95845 | KC019079 |
| 95884 | KC019109 |
| 95636 | KC018891 |
| 95872 | KC019101 |
| 95446 | KC018713 |
| 95840 | KC019074 |
| 95279 | KC018561 |
| 95335 | KC018611 |
| 95414 | KC018684 |
| 95425 | KC018693 |
| 95695 | KC018946 |
| 95350 | KC018626 |
| 95815 | KC019056 |
| 95310 | KC018588 |
| 95603 | KC018863 |
| 95598 | KC018858 |
| 95542 | KC018802 |
| 95421 | KC018689 |
| 95308 | KC018586 |
| 95644 | KC018899 |
| 95251 | KC018534 |
| 95433 | KC018701 |
| 95706 | KC018955 |
| 95653 | KC018910 |
| 95709 | KC018958 |
| 95267 | KC018550 |
| 95336 | KC018612 |
| 95382 | KC018655 |
| 95249 | KC018533 |
| 95768 | KC019014 |
| 95637 | KC018892 |
| 95808 | KC019050 |
| 95547 | KC018805 |
| 95531 | KC018791 |
| 95323 | KC018600 |
| 95564 | KC018818 |
| 95303 | KC018581 |
| 95533 | KC018793 |
| 95248 | KC018531 |
| 95635 | KC018890 |
| 95412 | KC018681 |
| 95503 | KC018769 |
| 95841 | KC019075 |
| 95513 | KC018778 |
| 95689 | KC018940 |
| 95543 | KC018803 |
| 95457 | KC018722 |
| 95856 | KC019088 |
| 95814 | KC019055 |
| 95684 | KC018935 |
| 95486 | KC018755 |
| 95468 | KC018733 |
| 95721 | KC018971 |
| 95238 | KC018523 |
| 95781 | KC019027 |
| 95619 | KC018877 |
| 95285 | KC018566 |
| 95289 | KC018569 |
| 95738 | KC018986 |
| 95599 | KC018859 |
| 95226 | KC018510 |
| 95839 | KC019073 |
| 95207 | KC018487 |
| 95265 | KC018547 |
| 95628 | KC018886 |
| 95790 | KC019036 |
| 95245 | KC018529 |
| 95436 | KC018704 |
| 95210 | KC018489 |
| 95715 | KC018965 |
| 95287 | KC018568 |
| 95852 | KC019084 |
| 95339 | KC018615 |
| 95364 | KC018638 |
| 95393 | KC018666 |
| 95696 | KC018947 |
| 95443 | KC018710 |
| 95363 | KC018637 |
| 95660 | KC018916 |
| 95611 | KC018869 |
| 95495 | KC018762 |
| 95727 | KC018976 |
| 95631 | KC018888 |
| 95460 | KC018725 |
| 95563 | KC018817 |
| 95826 | KC019064 |
| 95639 | KC018894 |
| 95522 | KC018785 |
| 95805 | KC019048 |
| 95519 | KC018782 |
| 95799 | KC019044 |
| 95784 | KC019030 |
| 95788 | KC019034 |
| 95312 | KC018591 |
| 95737 | KC018985 |
| 95281 | KC018562 |
| 95333 | KC018609 |
| 95241 | KC018526 |
| 95354 | KC018629 |
| 95757 | KC019004 |
| 95789 | KC019035 |
| 95730 | KC018978 |
| 95541 | KC018801 |
| 95548 | KC018806 |
| 95331 | KC018607 |
| 95763 | KC019009 |
| 95507 | KC018772 |
| 95270 | KC018554 |
| 95847 | KC019081 |
| 95526 | KC018788 |
| 95423 | KC018691 |
| 95338 | KC018614 |
| 95497 | KC018764 |
| 95435 | KC018703 |
| 95678 | KC018931 |
| 95320 | KC018598 |
| 95498 | KC018765 |
| 95802 | KC019046 |
| 95516 | KC018779 |
| 95759 | KC019006 |
| 95380 | KC018653 |
| 95720 | KC018970 |
| 95575 | KC018832 |
| 95711 | KC018960 |
| 95661 | KC018917 |
| 95643 | KC018898 |
| 95832 | KC019068 |
| 95476 | KC018742 |
| 95218 | KC018501 |
| 95761 | KC019008 |
| 95462 | KC018727 |
| 95671 | KC018924 |
| 95888 | KC019113 |
| 95604 | KC018864 |
| 95551 | KC018808 |
| 95835 | KC019070 |
| 95658 | KC018914 |
| 95574 | KC018831 |
| 95299 | KC018577 |
| 95535 | KC018795 |
| 95629 | KC018887 |
| 95698 | KC018949 |
| 95408 | KC018678 |
| 95685 | KC018936 |
| 95801 | KC019045 |
| 95654 | KC018911 |
| 95822 | KC019061 |
| 95243 | KC018528 |
| 95576 | KC018833 |

**Reference:**

Kamali, A., M. A. Price, S. Lakhi, E. Karita, M. Inambao, E. J. Sanders, O. Anzala, M. H. Latka, L. G. Bekker, P. Kaleebu, G. Asiki, A. Ssetaala, E. Ruzagira, S. Allen, P. Farmer, E. Hunter, G. Mutua, H. Makkan, A. Tichacek, I. K. Brill, P. Fast, G. Stevens, P. Chetty, P. N. Amornkul, J. Gilmour and I. A. H. P. Partnership (2015). "Creating an African HIV Clinical Research and Prevention Trials Network: HIV Prevalence, Incidence and Transmission." PLoS One **10**(1): e0116100.
